# The impact of rising temperatures on water balance and phenology of European beech (*Fagus sylvatica* L.) stands

**DOI:** 10.1007/s40808-019-00602-1

**Published:** 2019-05-22

**Authors:** Klaus Dolschak, Karl Gartner, Torsten W. Berger

**Affiliations:** 1 Department of Forest- and Soil Sciences, Institute of Forest Ecology, University of Natural Resources and Life Sciences (BOKU), Peter Jordan-Straße 82, 1190 Vienna, Austria; 2 Department of Forest Ecology and Soil, Federal Research and Training Centre for Forests, Natural Hazards and Landscape, Seckendorff-Gudent-Weg 8, 1131 Vienna, Austria

**Keywords:** Forest water balance, Box model, Soil drought, Climate change, Beech phenology, Simulated annealing

## Abstract

In this article, we outline the set-up and the application of an eco-hydrological box model, with the aim to describe the water balance of deciduous (*Fagus Sylvatica* L.) forest stands. The water balance model (WBM) uses standard meteorological parameters as input variables and runs on a daily time step. It consists of two modules. The aboveground module (1) comprises routines for fog precipitation generation, precipitation interception and snowfall/snowmelt dynamics. Covered belowground processes (2) are bypass flow, percolation, soil evaporation and transpiration, where the latter two processes are considered separately. Preceding to the WBM, a routine is introduced, specifying the intra-annual foliage dynamics of beech. Emphasis is also laid on the inter-annual variation of beech phenology. Leaf sprouting and leaf senescence are calculated as functions of day-length and air temperature. The WBM was applied to four European beech dominated forest stands in the northeastern part of Austria. They are located on a gradient of declining annual precipitation (from west to east). The two easterly sites are located close to the (dry) limit of the natural distribution of beech. Records of soil moisture were used for the adjustment of 26 parameters. On all sites the calibration process (simulated annealing) delivered good predictions of soil moisture (Nash–Sutcliffe efficiency≥ 0.925). Then, the obtained parameterization was used to apply different scenarios of global warming. The temperature was increased step-wisely up to 4 °C. All scenarios were run (1) with present phenological conditions and (2) with phenology responding to higher temperatures. This way, we wanted to assign the effect of higher temperatures and longer growing seasons on the water dynamics of the forest stands. A warming of 1 °C corresponded roughly to an elongation of the growing season of 4.5 days, where the start of the growing season was affected more strongly than the end. Apparently, higher temperatures led to drier soils. The strongest change was observed in early summer, also amplified by an earlier start of the growing season. Rising temperatures led to lower export fluxes of liquid water, simultaneously increasing evapotranspiration (ET). The gain in ET was almost entirely assignable to increased soil evaporation. Drier soils led to a sharp depression of transpiration during summer months. This decline was compensated by the effect of elongated growing seasons. The risk of severe drought was increased by higher temperatures, but here the contribution of growing season length was negligible. Drier soils seem to hamper the stands’ productivity. For all warming scenarios, the estimated increase of the gross primary production, caused by longer periods of assimilation, is nullified by the effect of soil water deficit in mid-summer.

## Introduction

Climate change is assumed to have a strong impact on Central European forest ecosystems. Over the last 140 years, South Europe and the Alps experienced a temperature increase of 2 °C (Mayer et al. 2005). Current climate estimations point to a further rise of the global surface temperature of 2 °C in the next 40 years (Field et al. 2014); it seems likely that the alpine region will experience a temperature elevation which will even be stronger (EEA 2015). Beech is a dominant tree species in Central and Western European forests (Dittmar et al. 2003); the natural distribution is associated to the Atlantic to Sub-continental Climate (Sutmöller et al. 2008). As a species with a broad eco-physiological amplitude, it seems adequately adapted to resist climate change in the (humid and cool) Atlantic areal of distribution (Kölling et al. 2007). Especially on the southern limit of the species’ distribution a different picture is expected. There, the occurrence of beech is mainly restricted by the soil water availability (Ellenberg and Leuschner 1996). European Beech is a species which is particularly vulnerable to soil drought (Bolte et al. 2009). Dry and hot conditions have been known to restrict net primary production of beech forests significantly (Ciais et al. 2005). Higher temperatures are assumed to increase the frequency and intensity of soil drought due to the forcing effect on potential evapotranspiration (Bergh et al. 2003). In contrast to rising temperatures, annual precipitation sums are assumed to retain the present level, but there might be a shift in the seasonal pattern. Current estimations point towards increasing late-winter to spring precipitation, hand in hand with decreased precipitation during summer months (Geßler et al. 2007; Kunstmann et al. 2004), exacerbating soil water deficit.

Warmer conditions will lead to a temporal elongation of the growing season of beech (Vitasse et al. 2009). Under optimal conditions this would result in an increased productivity (Lindner et al. 2010). Under water limitation the opposite effect seems possible. High temperatures in spring favor growth at first. Later in the season they accelerate the soil water depletion, resulting in a sharp drop of carbon fixation by mid-summer (Dittmar et al. 2003; Kljun et al. 2007; Richardson et al. 2013).

A future increase of frequency and duration of drought periods during the growing season might alter the productivity, competitive and regenerative abilities of beech stands, especially on shallow soils (Geßler et al. 2007; Rennenberg et al. 2004). On these sites, it seems likely that beech stands will be replaced by drought resilient Oak-Hornbeam forest associations (Theurillat and Guisan 2001).

In this work, we set up a model describing the water balance of deciduous forest stands. The routing of modeled water fluxes is illustrated in Figure S1 of the supplementary. Due to a strong connection of processes such as light extinction, precipitation interception or the stands water demand to the stands leaf area (van Wijk and Williams 2005), we see the need to describe the temporal dynamics of the vegetation cover. Preceding to the WBM, a phenological routine is introduced, consisting of 2 elements: (1) the calculation of inter-annual variations of leaf emergence and leaf senescence, and (2) a quantitative measure, describing the stands seasonal development of the leaf area.

This way we assessed the soil moisture dynamics of four beech stands, which are located in the north-easterly part of Austria, close to the dry distribution limit of European beech. We analyzed the effect of climate change on the sites’ water balance. In that context, possible changes in CO_2_ air concentration or the precipitation pattern were neglected; the focus lay solely on the impact of rising temperatures on the stands’ soil moisture regime. This way, we assessed temperature driven changes of the frequency and intensity of soil water deficit. At last we tried to identify factors which were influencing the stands’ vulnerability and resilience towards soil drought.

## Materials and methods

### Study sites

**Fig.1:**
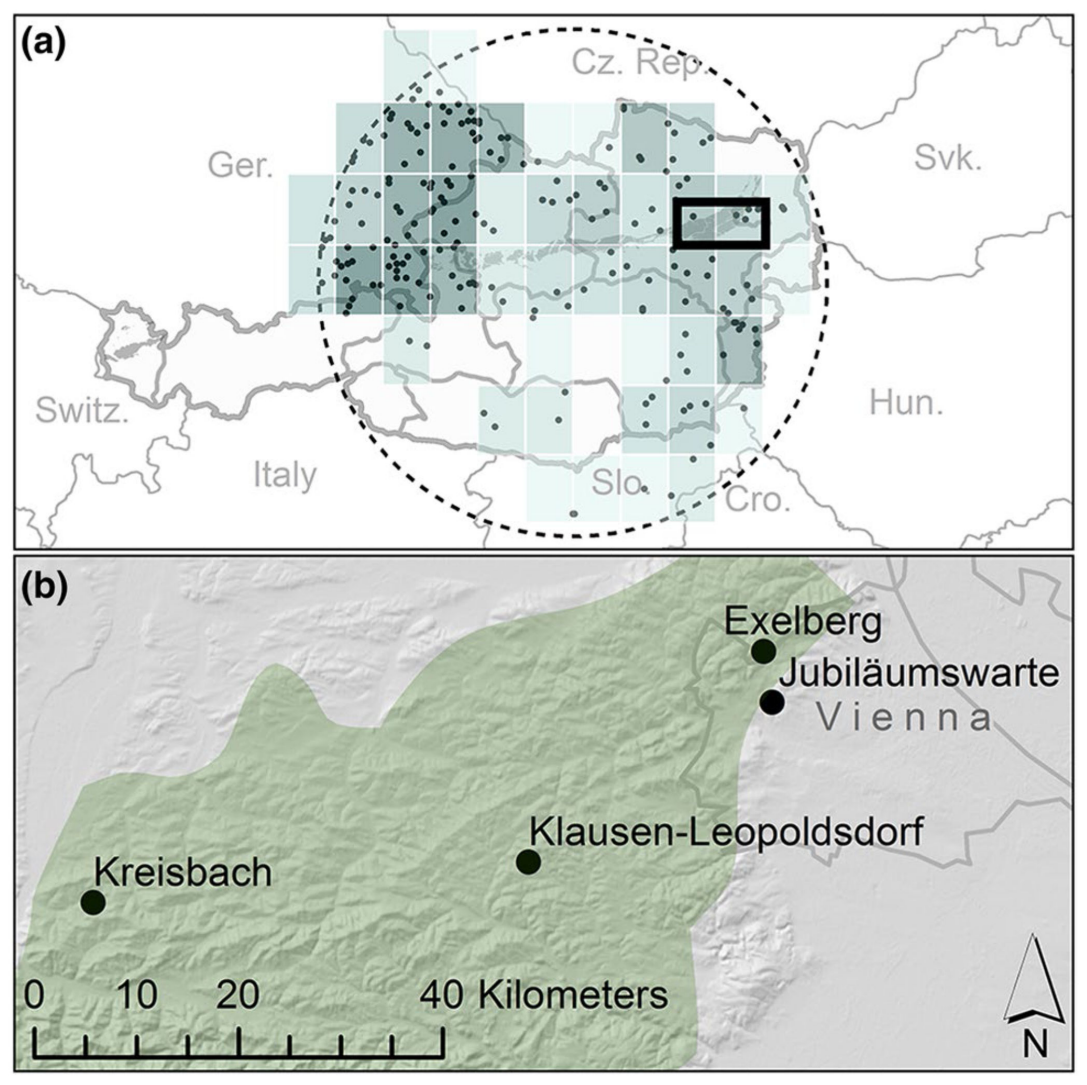
**a** For calibration of the phenological model, PEP725 (Pan European Phenology Data) beech stands (small black dots) were accessed within a radius of 200 km centered to 47°42′00″N, 14°30′00″E. One average time series of air temperature was calculated, using data from the E-OBS gridded dataset (Tank et al. 2002b) (0.5° resolution, turquoise rectangles). The frequency of selected sites within one grid cell defines the relative weight, the cell receives in the calculation of the average; darker cells correspond to higher weightings. **b** Locations, used in the calibration of the WBM (black dots). All sites are beech dominated stands and share their geological bedrock (flysch). The green area represents the natural distribution of European Beech according to the Map of the Natural Vegetation of Europe (Bohn et al. 2004)

The investigated forest stands are located in the north-easterly part of Austria in the foothills of the Northern Calcareous Alps (see Fig. 1b). The parent material for soil formation is Flysch, which consists of old tertiary and mesozoic sandstones and clayey marls of maritime origin. Due to high clay content, the saturated hydraulic conductivity is low, leading to frequent episodes of waterlogging. Therefore, the soil type can be classified as stagnic cambisol according to the WRB soil classification (IUSS Working Group 2006) throughout all studied sites. The mean annual temperature in the study area is approximately 9 °C. Precipitation declines from west to east, with average annual sums ranging from 820 mm (Kreisbach) to 652 mm (Vienna).

In the framework of the International Co-operative Programme on Assessment and Monitoring of Air Pollution Effects on Forests (ICP Forests), the Austrian Research Centre for Forests operates several intensively monitored forest sites (Level II) (Neumann et al. 2001). In addition to other environmental parameters, meteorological conditions are monitored continuously. Soil moisture (Campbell CS615 FDR probe) is recorded at 3 different depths (15, 30, 60 cm).

The model was originally set up on data from the Level II plot Klausen–Leopoldsdorf (KL), which is located in the Vienna Woods (48°07′16″N, 16°02′52″E), at an elevation of 510 m a. s. l. The research site is a pure beech (*Fagus sylvatica* L.) stand, which was planted in the late thirties of the last century. The site is facing NE with an inclination of 20%. The actual forest vegetation coincides with the potential natural one and can be classified as *Hordelymo*-*Fagetum* (Mucina et al. 1993). For a more detailed site description see Neumann et al. (2001).

The Kreisbach (KB) site, which is located south of St. Pölten (48°05′50″N, 15°39′50″E) at an elevation of 470 m a. s l., is a mixed European beech-Norway spruce (*Picea abies* L.) stand, with beech dominating. The stand is facing NNE with an inclination of 19%. The natural plant association can be classified as *Asperulo odoratae*-*Fagetum*. From 1998 to 2003 the site was monitored meteorologically within the framework of a special research program on Forest Ecosystem Restoration. Soil moisture records (Trase1 TDR probe) exist for 4 different depths (10, 20, 40, 55 cm). For a more detailed site description see Schume et al. (2003).

The third site (Jubiläumswarte, JU) is located within the municipal area of Vienna at the eastern edge of the Vienna Woods (48°13′12″N, 16°15′56″E), at an elevation of 440 m a. s. l. The site, which is a pure beech stand with an estimated age of 125-150 years, is facing SSE with an inclination of 15%. As a matured stand it is showing signs of collapse but also strong natural regeneration. Different to the other investigated sites, the bedrock contains calcareous material, reflected in higher base saturation and soil pH.

The Exelberg (EX; 48°14′40″N, 16°15′18″E) site is located in Lower Austria close to the border to Vienna, 2.8 km northwest of Jubiläumswarte. This site is also a pure beech stand. We estimated the stands’ age approximately 100 years. The site is also facing SE with an inclination of 22%. Two years of bi-weekly observations of soil moisture exist for 10, 30 and 60 cm depth. The latter two sites are located at the dry distribution limit of beech (see Bohn et al. 2004). They receive significantly less precipitation than the first two. Due to their location at upper hill slopes and their exposition, we see them prone to soil drought.

### Data sources

The WBM, which runs on a daily time step, uses standard meteorological data on a daily base as input. Time series of minimum (*T*_min_, °C), mean (*T*_mean_) and maximum (*T*_max_) temperature, the daily averages of relative humidity (rH), global radiation (gR, wm^−2^), and wind speed at 2 m above ground (*u*_2_, ms^−1^), as well as the observed 24-h precipitation sum (*P*_obs_, mm) are required. The phenological module utilizes daily *T*_mean_ and *T*_max_.

For gap filling purposes, data were accessed from the Austrian Meteorological Agency (ZAMG) as well as from the Austrian Hydrographic Service (eHYD). For the EX site, we accessed precipitation records from a private weather station. Missing data were replaced, using simple regression techniques, with data from highly correlated, neighboring stations.

For calibration of the phenological module, data were retrieved from the PEP725 database (PEP725 Pan European Phenology Data, data set accessed on 06/06/2015 at http://www.zamg.ac.at/pep725/). Two phenological stages were considered. (1) BBCH-11: leaf unfolding (LU) on the first visible leaf stalk, represents the onset of the growing season. (2) BBCH-94: autumnal leaf coloring (50% of leaves colored) (LC), marking the end of the growing season. In this work, phenological phases are calculated as functions of day-length and air temperature. Therefore, the gridded E-OBS dataset (a daily gridded observational dataset for meteorological parameters) was accessed (0.5°, regular grid), provided by the European Climate Assessment (ECA&D) (Tank et al. 2002a).

Due to strong site variations of phenological events, the set-up of the phenological model was conducted, utilizing multiple phenological sites within a radius of 200 km, centered to 47°42′00″N, 14°30′00″E (see Fig. 1a). The phenological dataset was scanned for outliers using Tukey’s test. Parallel data was checked for month-mistakes (Schaber and Badeck 2002). Only time series with 10 or more annual observations were considered in the calculation. To overcome site specific effects, the influence of phenotypic plasticity (Capdevielle-Vargas et al. 2015), or divergences in the assessment of phenological stages (Estrella and Menzel 2006), the calibration of the model was performed on an assembled time-series. To generate this assembled time-series, we implemented the 3rd method which was proposed in Häkkinen et al. (1995). To each DoY of each time-series a site wise offset (*O_s_*) was applied. The aim was minimizing the residual between site-wise time-series and the mean time-series over all sites:

(1) $$\underset{O_{\text{s}}}{\min}\;[{\sum_{y}\sum_{s}\left(\left({\text{DoY}}_{sy}-O_{s}\right)-\frac{\sum_{s}\left({\text{DoY}}_{sy}-O_{s}\right)}{n_{y}}\right)}^{2}].$$

To achieve this, we used the classical hill climber algorithm. 40,000 iterations were used to adapt *O_s_* for all considered sites. The sum of squared residuals could be reduced to approximately 55% of its initial value. To ensure that the residual sum equals zero, the overall mean before and after the optimization was calculated; the difference between both means stated a second offset which was applied to DoY_*sy*_:

(2) $$o_{n}=-\frac{\sum_{sy}O_{s}}{n_{sy}}.$$

Parallel, a time-series of the average *T*_min_, *T*_mean_ and *T*_max_ was calculated over all E-OBS grid cells, comprising selected phenological sites, whereat the number of sites within the cell defined the relative weighting the cell received in the calculation of the average.

### Model description

#### Annual phenological key events

Beech can be considered a late flushing species (Vitasse and Basler 2013). By that, it is following a rather conservative strategy, aiming to decrease the risk of late frost exposure (Caffarra and Donnelly 2011; Körner and Basler 2010). There are several environmental signals involved, in the triggering of the start of the growing season. Of high relevance is the seasonal course of the photoperiod (Basler and Körner 2012), meaning the day length has to exceed a critical threshold in spring before bud burst might occur (Körner and Basler 2010). According to Laube et al. (2014), an environmental trigger which is weighted even more strongly, is the chilling demand, meaning winter temperatures, undershooting a threshold for a certain time, are promoting dormancy release in spring. Furthermore, leaf sprouting is accelerated by high spring temperatures (Caffarra and Donnelly 2011; Vitasse et al. 2009).

Compared to spring phenology, the environmental triggering of beech senescence is less understood (Estrella and Menzel 2006; Vitasse et al. 2009). Especially the role of temperature is discussed controversially. For European beech stands, Estrella and Menzel (2006) reported positive correlation of the August and September mean air temperature, with the date of leaf coloring. Surprisingly, the authors found also a negative correlation with temperature in May and June, meaning low average temperatures in late summer and high temperatures in late spring promote the temporal occurrence of leaf senescence. Whether the latter was a direct temperature effect, or the effect of (temperature correlated) drought during critical phenological stages, was not examined.

In this section, a model is presented, describing the onset of the growing season as function of daily air temperature. Assuming the chilling demand generally over-satisfied for central European forest stands (Fu et al. 2012), only the forcing effect of air temperature is considered.

A common approach to quantify the forcing effect of air temperature on spring development requires the definition of a threshold temperature; below this temperature no forcing is taking place, above the temperature forcing is assumed proportional to the temperature difference between actual and threshold temperature (Cannell and Smith 1983). To achieve a more gradual transition of the forcing response to air temperature, a piecewise combination of a first and second order polynomial is presented in this work. The full formulation of the function, which is optically resembling the shape of a hockey stick, is stated in the “Appendix” (Eqs. 18, 23, 24). Below the threshold temperature (*T*_0,LU_) the response is assumed 0. The onset is described with a 2nd order polynomial. A second key temperature (*T*_1,LU_) defines the transition from quadratic to linear response, where *m*_LU_ sets the forcing rate at *T*_1,LU_. Most approaches for predicting spring phenology as a function of air temperature make use the daily mean temperature. In this work, it was found that the average of daily *T*_mean_ and *T*_max_, aiming to represent the average daytime temperature (*T*_day_), displayed higher force of expression in the prediction of LU:

(3) $$f_{T}=\text{hockey}\left(T_{\text{day}},T_{0,\text{LU}},T_{1,\text{LU}},0,m_{\text{LU}}\right).$$

Analogue to Blümel and Chmielewski (2012), a day length term is included, accounting for the photoperiodic influence on spring development. The day length (*dl*, hours) was calculated as function of the day of the year (DoY) and the geographical latitude, analogue to Swift (1976). A model parameter in the exponent (*x*_LU_) adds one degree of freedom. Preventing vast values in the photoperiod term, day length is normalized by dividing by 14 h:

(4) $$f_{dl}={\left(\frac{dl}{14h}\right)}^{x_{\text{LU}}}.$$

The daily forcing is described as the product of a function of air temperature and day length:

(5) $$f_{\text{LU}}=f_{T}f_{dl}.$$

The temperature accumulation starts with DoY_0,LU_. LU is triggered after the accumulation of 10 forcing units:

(6) $$Sf_{\text{DoY1,LU}}=\sum_{{\text{DoY}=\text{DoY}}_{0,\text{LU}}}^{{\text{DoY}}_{1,\text{LU}}}f_{\text{LU},\text{DoY}}=10.$$

The calibration of the model was conducted on the assembled time-series, described in the previous section. The phenological data which used in this work was provided in discrete daily resolution, but calculating means over several sites led to non-integer values for the day of the year of the phenological event. A model, which is treating phenological events as discrete in time, cannot overcome the residual caused by the decimal places. To surmount this minor but unnecessary flaw, another function is introduced: The difference of the sum, necessary to trigger budburst and the sum of the day prior to budburst, divided by the difference of the sum, achieved on the budburst day and the prior day, minus a half day is calculated:

(7) $$c_{\text{LU}}=\frac{10-Sf_{\text{DoY}1,\text{LU}\;‐\;1}}{Sf_{\text{DoY}1,\text{LU}}-Sf_{\text{DoY}1,\text{LU}\;‐\;1}}-\frac{1}{2}.$$

A distinct exceeding of the temperature sum, necessary to trigger the event, on DoY_1,LU_ will result in a negative value of c_LU_, Therefore, DoY_LU_ will be shifted to a slightly earlier point of time. A weak overshooting will result in a delay of the event. The DoY of leaf unfolding is finally calculated:

(8) $${\text{DoY}}_{\text{LU}}={\text{DoY}}_{1,\text{LU}}+c_{\text{LU}}$$

The approach for modeling the annual variability of the end of the growing season, is based on the findings of Estrella and Menzel (2006). A linear model is set up, utilizing averaged *T*_mean_ of 2 seasonal periods (DoY_0,LC_ – DoY_1,LC_, DoY_2,LC_ – DoY_3,LC_): (1) late spring and (2) late summer–early autumn. Within these periods, a parabolic function assigns weight (*w*_LC_) to the observed *T*_mean_:

(9) $$w_{\text{LC}}=\{\begin{array}{ll} 0, & \text{DoY}\leq {\text{DoY}}_{0,\text{LC}}\\ 4h_{\text{LC}}\frac{\left({\text{DoY}}_{1,\text{LC}}-\text{DoY}\right)\left({\text{DoY}-\text{DoY}}_{0,\text{LC}}\right)}{{\left({\text{DoY}}_{1,\text{LC}}{-\text{DoY}}_{0,\text{LC}}\right)}^{2}}, & {\text{DoY}}_{0,\text{LC}}<\text{DoY}<{\text{DoY}}_{1,\text{LC}}\\ 0, & {\text{DoY}}_{1,\text{LC}}\leq \text{DoY}\leq {\text{DoY}}_{2,\text{LC}}\\ 4\frac{\left({\text{DoY}}_{3,\text{LC}}-\text{DoY}\right)\left({\text{DoY}-\text{DoY}}_{2,\text{LC}}\right)}{{\left({\text{DoY}}_{3,\text{LC}}{-\text{DoY}}_{2,\text{LC}}\right)}^{2}} & {\text{DoY}}_{2,\text{LC}}<\text{DoY}<{\text{DoY}}_{3,\text{LC}}\\ 0 & \text{DoY}\geq {\text{DoY}}_{3,\text{LC}} \end{array}\;,$$

Due to the fact that senescence dates correlate negatively with *T*_mean_ in late spring, the parabolic function in the first period yields negative values, with a minimum of *h*_LC_. The weighted average *T*_mean_ (*wA*_LC_) inside the temporal window is calculated. Figure S2 in the supplementary information states a graphical representation of the assessment of spring and autumn phenology:

(10) $$wA_{\text{LC}}=\frac{\sum_{i={\text{DoY}}_{3\text{LC}}}^{{\text{DoY}}_{0\text{LC}}}T_{\text{mean},i}w_{\text{LC},i}}{\sum_{i={\text{DoY}}_{3\text{LC}}}^{{\text{DoY}}_{0\text{LC}}}\left|w_{\text{LC},i}\right|}.$$

The annual DoY of LC is then calculated in a linear equation:

(11) $${\text{DoY}}_{\text{LC}}=k_{\text{LC}}wA_{\text{LC}}+d_{\text{LC}}.$$

The functions to determine LU and LC were optimized, using a combination of simulated annealing (Kirkpatrick 1984) and the Gauss–Newton algorithm. Performance criterion was the Nash–Sutcliffe efficiency (NSE) (Nash and Sutcliffe 1970):

(12) $$\text{NSE}=1-\frac{\sum_{i=1}^{n}{\left({\text{obs}}_{i}-{\text{sim}}_{i}\right)}^{2}}{{\sum_{i=1}^{n}\left({\text{obs}}_{i}-\bar{\text{obs}}\right)}^{2}}.$$

To express the model error in days, the root mean squared error is also calculated:

(13) $$\text{RMSE}={\left[n^{-1}\sum_{i=1}^{n}{\left({\text{obs}}_{i}-{\text{sim}}_{i}\right)}^{2}\right]}^{\frac{1}{2}}.$$

#### Water balance model

An exhaustive description of the setup and the formulation of the WBM can be found in the supplementary!

### Model application

The simulator was parameterized, using time-series of observed soil moisture. We used records of different depths to calculate a mean time-series, aiming to reflect the integrated volumetric soil moisture over soil depth (*z*_r_). Averages over a soil depth of 500 mm were calculated for all sites. Both canopy and the snowpack storage were initialized at 0 mm. Soil water storage was initialized at the product of soil depth and the water content at field capacity (*z*_r_*θ*_fc_). The model ran a 200 day spin up, prior to the performance analysis timeframe. Twenty-six parameters (compare Table 3) were optimized by inverse modeling; a simulated annealing algorithm was applied. Performance criterion was the Nash–Sutcliffe efficiency (Eq. 12). The parameterization was performed over the entire investigation timeframe (Table 2).

### Water stress assessment

The obtained parameterization was used to run the model over a reference climate period of 30 years. The time-frame was set from Jan 1983 to Dec 2012. Then, eight scenarios of climate warming were applied. Temperatures were increased from 0 to 4 °C in one degree steps. Four scenarios were run under the assumption that (1) phenology retains the values of the reference climate; four scenarios were run with (2) phenology responding to warmer conditions. This way, we wanted to quantify the influence of elongated growing seasons on the stands’ water consumption and soil water deficit.

Different levels of water stress were calculated. The transpiration index (*T*_i_) states the daily ratio between simulated actual transpiration and potential transpiration (transpiration which would occur under optimal root water supply) (Clausnitzer et al. 2011; Vilhar 2016). In our formulation, it corresponds to the water stress coefficient (Kc_s,tree_) in the calculation of the actual transpiration rate:

(14) $$T_{\text{i}}={\text{Kc}}_{\text{s,tree}}$$

A level of one corresponds to unlimited transpiration, a level of zero would correspond to a complete shutdown of transpiration. Investigating beech stands (Schwärzel et al. 2009) found indications of noteworthy water stress when *T*_i_ fell below 70%. Therefore, we set the threshold for (at least) moderate soil water deficit to 0.7. According to Bréda et al. (2006), xylem embolism occurs when stomatal conductance drops below 10% of its initial value. Therefore, a second stress level was calculated: If *T*_i_ falls below 0.1, we consider the stand affected by severe drought.

According to Granier et al. (1999), water stress occurs when the relative extractable water content (REW) drops below the critical value of 0.4. REW is calculated by normalizing theta to the interval from the wilting point to field capacity. The formulation, which is presented here, allows soil moisture below the wilting point (*θ*_pwp_) and above field capacity (*θ*_fc_). Therefore, REW can take values below zero and above 1!

(15) $$\text{REW}=\frac{\theta -\theta_{\text{pwp}}}{\theta_{\text{fc}}-\theta_{\text{pwp}}}$$

Then, the number of days during the growing season with *T*_i_ or REW below the defined threshold was calculated. In this assessment, we considered the growing season as interval from the 25th of March (DoY = 84) to the 11th of November (DoY = 315). Years with more than 120 growing season days of *T*_i_ > 0.7 were defined as dry years. The threshold for drought years was reached with a minimum of 31 growing season days with *T*_i_ below 0.1. At last, to gain information about the stands’ photosynthetic activity, we estimated the gross primary production (GPP, gm^−2^d^−1^) as the product of water-use efficiency (WUE) and the transpiration rate:

(16) $$\text{GPP}=E_{\text{C}}\text{WUE}$$

To estimate the WUE, we relied on an empirical relationship, which was proposed by Tang et al. (2006). The water-use efficiency was calculated as function of the vapor pressure deficit (VPD):

(17) $$\text{WUE}=4.4+15.69{\text{e}}^{-5.94\text{VPD}}.$$

## Results and discussion

### Timing of phenological events

**Fig. 2:**
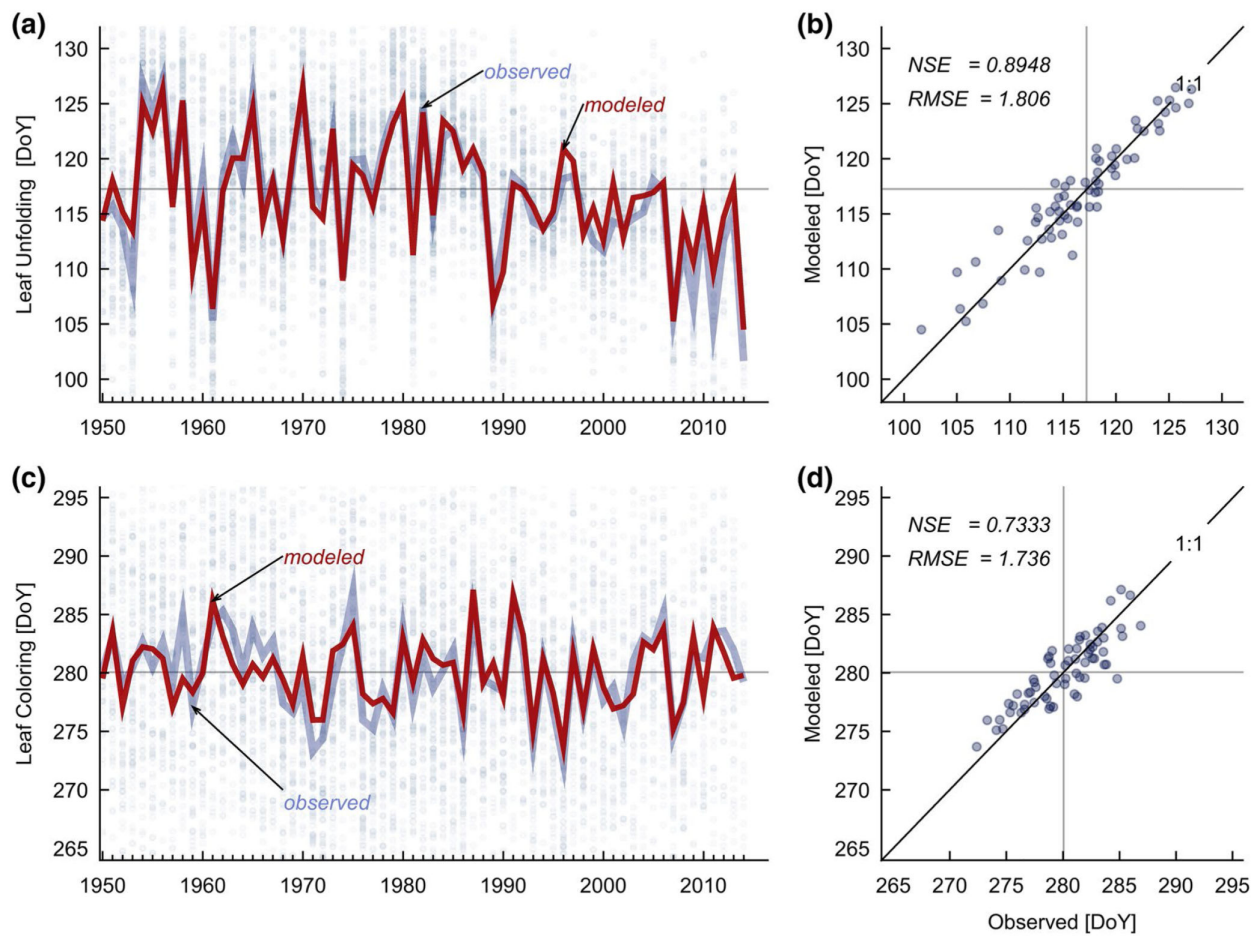
**a**, **c** The model was fitted to an assembled time-series of annual DoYs (pale solid lines), which were calculated, if more than 10 annual observations (small dots) were available. Leaf unfolding and coloring data were processed analogously (**a**) where LU clearly shows a trend towards earlier onsets of the growing season in last decades, LC (**c**) reveals no such pattern. The reason for this might be found in the counteracting effect of late spring and late summer temperatures. **b**, **d** Observed assembled time-series mean of leaf unfolding and leaf coloring vs. the modeled timing of the event. Details about calculations of Nash–Sutcliffe efficiency (NSE) and root mean squared error (RSME in days) are given in the text

Both, spring and autumn phenology showed high intra-annual plasticity. Nevertheless, after transforming the data to an assembled time-series, LU revealed a distinct pattern, with a recent trend towards an earlier onset of the growing season (see Fig. 2a–d). On the contrary to very high intra-annual plasticity, the year to year variations of the winsorized means of LC were smaller compared to LU. The reason no trend towards a delaying of senescence was observed, might be found in the counteracting effect of late spring and late summer temperatures.

The parameterization of the LU module led to a good fit between observed and modeled onset of the growing season (compare Table 1; NSE > 0.89, RMSE < 2 days). A very low modeled *T*_0,LU_ (< − 11 °C) seems to be non-meaningful in a plant physiological sense. On the other hand, the simulated effect of temperature forcing at cold conditions is partially nullified by low multiplier values from the day-length term, at the beginning of the forcing period, in early winter (see Fig. S5).

The regression approach, to predict the end of the growing season, utilizing the mean temperature of two temporal windows, was also suitable to reproduce the observed pattern to a sufficient degree (NSE > 0.73, RMSE < 2 days). The mechanism behind the acceleration of senescence by high temperatures in late spring was not elaborated in this work. Nevertheless, two explanatory assumptions are stated: High temperatures in spring point to an early onset of the growing season. This, and the high temperature itself might increase the water consumption, (1) inducing drought during critical phenological stages. (2) High temperatures in late spring might support the development of specific pest or pathogens, leading to stress induced, premature leaf coloring (see also Menzel et al. 2008).

### Water balance

The approaches to describe fog precipitation, precipitation interception, as well as the responses of evapotranspiration and percolation to soil moisture, presented in this work are novel. Therefore, their parameterization cannot be relied on the literature data. Due to unavailability of direct measurements, they were deduced by model optimization, applying broad search ranges. In cases where literature values existed (e.g. degree day factors for snowmelt, field capacity), parameter values were searched in the close proximity of values stated in the literature. On both investigated sites, the optimization process lead to a good fit between the observed and predicted soil water content (NSE < 0.92.5); the simulator was capable to track the temporal dynamics of the daily average soil moisture (*θ*, L L^−1^), over the whole investigation timeframe (Fig. S6) (Table 2).

In the following section, the parameterization of the WBM is discussed. For the parameter configuration of all 4 sites, see Table 3. On the sites KR and KL, the fog precipitation module had no improving effect on the models’ performance. In both cases, the optimization process led to fog coefficient (*f*_c_) values close to zero (see Table 3). The amount, fog is contributing to the total precipitation, seems insignificant at these locations. An explanation might be found in the sites relief; both investigated stands are located at lower hill slopes, partially shielded from (at least) two directions. A different picture was found on the EX and JU site; they are both located at upper hill slopes, close to the hill top, leaving them much more exposed to direct air flow.

The optimization process lead to relatively high parameters values, describing the maximum capacity of canopy storage (*C*_max_). For a central European beech stand, Gerrits et al. (2010) reported a canopy *C*_max_ ranging from 0.4 mm for winter conditions to 0.9 mm in summer. In this work, the parameterization led to a maximum storage capacity of 4.3 and 2.7 mm for the KB and the KL site, respectively. For the beech stand mentioned above, Gerrits et al. (2010) calculated a litter layer storage capacity with a yearly average of 1.8 mm, temporally peaking in autumn (2.8 mm). So it seems possible, that high *C*_max_ might be explained by the contribution of the litter layer to precipitation interception. Soil moisture records, utilized in the calibration process, existed for a minimum depth of 10 cm and 15 cm, for Kreisbach and Klausen-Leopoldsdorf, respectively. It seems also plausible that the canopy interception module accounts for soil water storage/interception, caused by the topmost layer of the mineral soil. An alternative to explain high values for *C*_max_ arises from the assessment of precipitation on a daily time-step: The possibility of multiple storms during 1 day is neglected. In such a case, our formulation might underestimate canopy storage (compare Pearce and Rowe 1981). Here, high values for *C*_max_ would have a compensating effect. High values for the coefficient, scaling the reference evapotranspiration (ET_O_) to canopy evaporation (Kc_canopy_), could be explained by the low surface resistance of the wet canopy (Herbst et al. 2008). The combination of high Kc_canopy_ and high *C*_max_ values is leading to high interception evaporation (*E*_I_) fluxes. On the KB site, deposition chemistry was monitored from the beginning of May 2002 to the end of October 2003 (Berger et al. 2008). In this course, also canopy evaporation fluxes were estimated as the difference between observed open area precipitation and the sum of throughfall and stemflow. A determined annual interception sum of 238 mm (26% of the open area precipitation) is in close resemblance to the estimate of the mean annual interception, presented in this work. On the KL site, throughfall was monitored in a bi-weekly interval from 2006 to 2010. For dormant and growing season, 5.8% and 11.9%, of the observed precipitation was intercepted by the canopy, respectively. For the same temporal interval, the simulation delivered canopy evaporation percentages of 8 and 11.9 for the dormant and growing season, respectively. For different beech stands, Peck and Mayer (1996) reported rainfall interception ranging from 5 to 48% of total precipitation, with a mean of 20%. On our investigated sites, modeled annual rainfall interception (canopy evaporation), was found on the low end of these estimates. Precipitation interception decreased from west to east, reaching its lowest value on the EX site (Table 4).

The calibration process lead to an unremarkable parameterization of the module, describing snow accumulation and snowmelt. Threshold temperatures for snowfall and snowmelt, as well as the degree day factors for snowmelt, were in the range of literature values. For a summary on threshold temperatures for snowfall, see Feiccabrino and Lundberg (2008), a summary on snowmelt degree-day factors for various catchments is given by Hock (2003).

On the KR forest location, an assessment of physical soil characteristics revealed a pore volume 52%, and a volumetric water content of 18.85% at 1 MPa (Schume et al. 2004). The parameterization for this site delivered values for saturation water content (*θ*_sat_) and *θ*_pwp_, which are located in the close vicinity of the measured ones.

For different beech stands, Peck and Mayer (1996) reported annual transpiration (*E*_C_) sums ranging from 268 to 601 with a mean of 363 mm. Our estimations of annual *E*_C_ were below these values, on all sites (Table 4). Especially on the KR and KL plot, is seems possible that transpiration water fluxes were slightly underestimated in the simulation outcome (Table 4). On the KR plot, beech roots were found at a soil depth of 85 cm (Schmid 2002). On the KL plot, an assessment of the sites soil characteristics revealed medium to strong root penetration down to 65 cm soil depth (Neumann et al. 2001). Due to reasons of soil moisture data availability, only the topmost 50 cm of the mineral soil were considered in this work, neglecting the possible contribution of deeper soil layers to the trees’ water supply.

On the KR and especially on the EX plot, the simulator delivered high relative fractions of bypass flow (Table 4). Analogue to the underestimation of *E*_C_ fluxes, the disregarding of the influence of deeper soil layers on the stands’ water balance might result in an overestimation of bypass flow.

High transpiration rates during the growing season, in contrast to low evaporative water consumption during the leafless period of the year, are leading to a distinct seasonal pattern of soil moisture. Where in the growing season, *θ* above field capacity occurs only exceptional, in the cold part of the year field capacity is rarely undershot, determining percolation through the soil profile as phenomenon of the dormant season (Fig. S7c,d).

### Climate change assessment

**Fig. 3:**
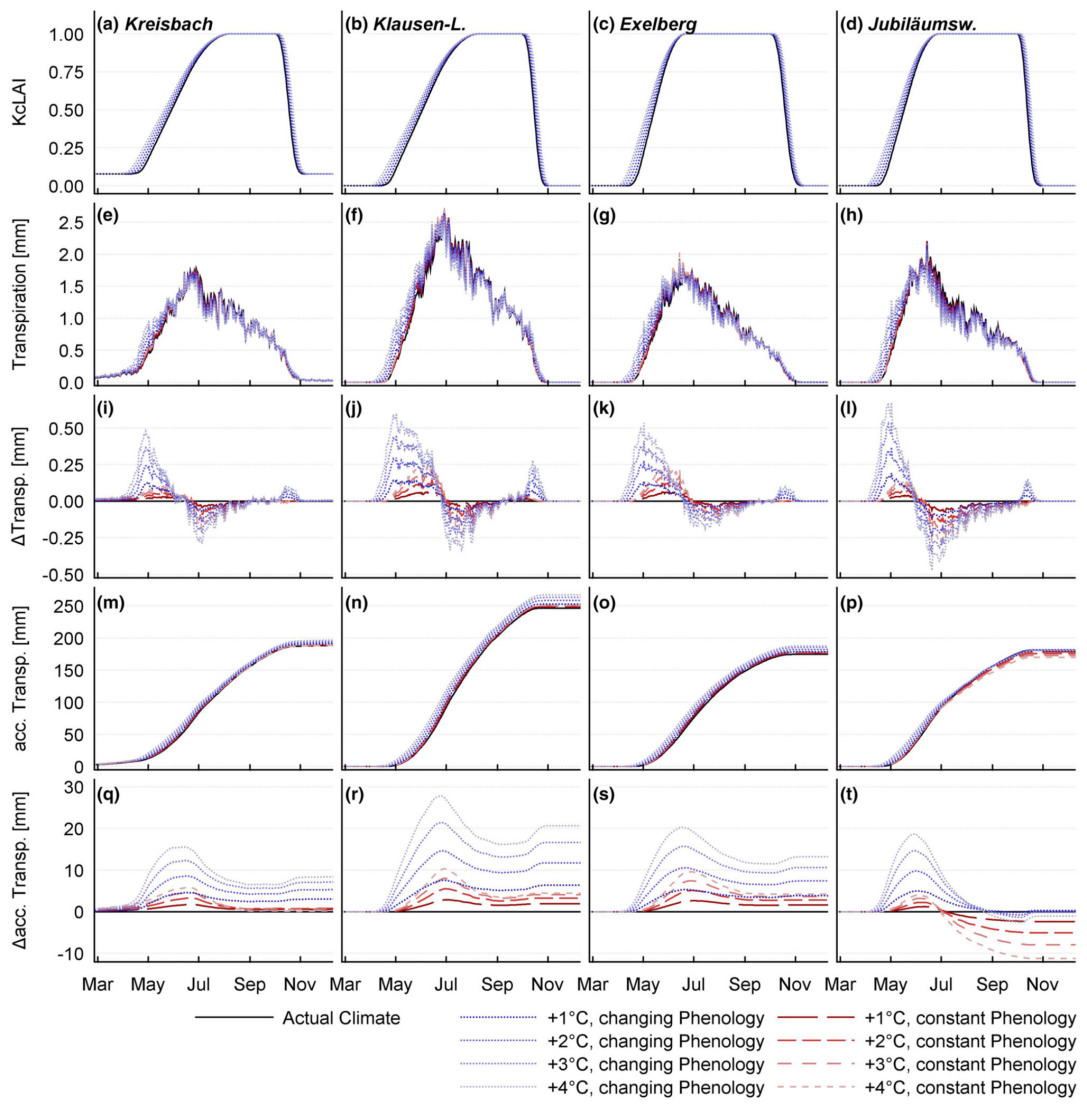
Result of the temperature sensitivity analysis on day of year base. **a**–**d** Relative leaf area: One degree temperature rise corresponds roughly to an increase of 4.5 days in growing season length, whereat LU is affected more strongly than LC. **e**–**h** Mean daily transpiration for all 4 sites: Transpiration is modeled highest at the beginning of summer. The KL site experiences the highest transpiration rates. **i**–**l** Change of daily transpiration compared to the actual climate: The increase of transpiration at the beginning of the growing season caused by higher temperatures and earlier LU is followed by a marked decrease in summer, due to soil water depletion. **m**–**p** Accumulated transpiration: The KL site displays the highest annual sums. **q**–**t** Change of the accumulated transpiration compared to the present climate. Without consideration of elongated growing seasons, the JU site responds with a decline of transpiration due to soil drought in mid-summer. This decline is compensated by the effect of the elongated growing seasons

Step-wisely increased temperatures led to a proportional elongation of the growing season. One °C roughly corresponded to an elongation of 4.7 days. The modeled relationship between warming and lengthening of the growing season was almost linear. All sites responded with a similar pattern (Fig. 3a–d). Spring phenology showed stronger reaction than autumn phenology. One °C warming corresponds to leaf unfolding, 3.5 days earlier. Regarding leaf senescence, the delaying effect of warmer late-summer temperatures was not fully compensated by the accelerating effect of high late-spring temperatures. A temperature increase of 1 °C corresponded to a delay of LC of 1.2 days.

**Fig. 4:**
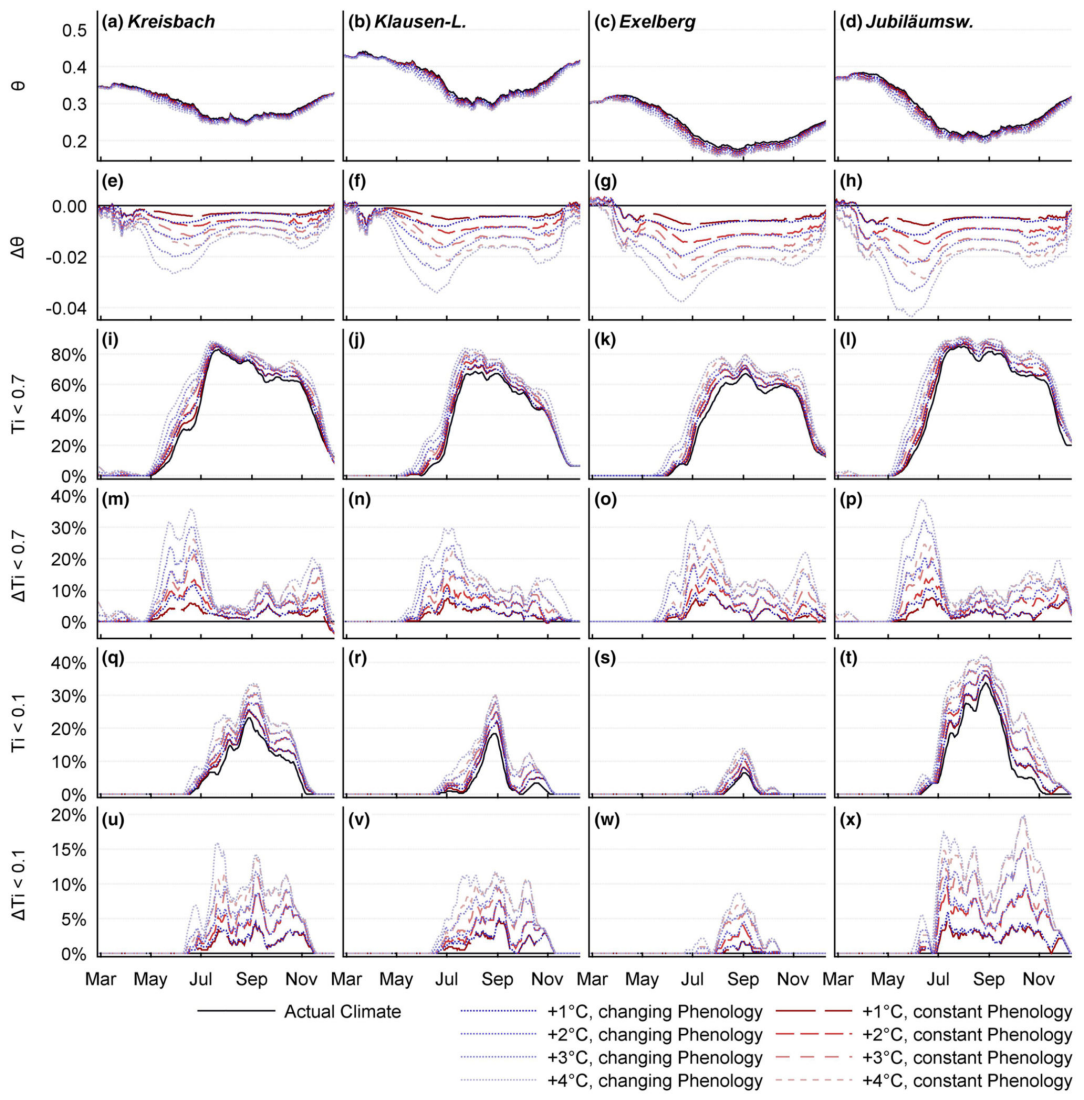
Result of the temperature sensitivity analysis on day of year base. **a**–**d** Seasonal course of the relative water content: All sites display the same seasonal dynamics with lowest soil moisture during the growing season. The EX site appears to be strikingly drier than the other sites. **e**–**h** Changes of soil moisture compared to the present climate: All investigated forest stands are apparently getting drier. The KB and KL site seem to be affected slightly weaker than the other sites. The reduction of soil moisture is most pronounced at the beginning of summer, also amplified by an earlier LU. **i**–**l** Probability of water stress (defined as *T*_i_ < 0.7): On all sites, water stress is the usual soil state during the warm season. The highest probability is found in mid-summer. **m**–**p** Change of the water stress probability, compared to the present state. Warmer temperatures e.g. longer growing seasons increase the risk of soil water stress, especially in early summer. An earlier LU amplifies the probability of water stress, especially at the beginning of summer. **q**–**t** Risk of severe soil drought (defined as *T*_i_ < 0.1): All sites show the highest probability of severe drought at the end of August. Although the EX site appears to be the driest site, the risk for severe water stress is strikingly low. The reason can be found in the low wilting point (see Table 3, Fig. S8), allowing transpirative water consumption at low soil moisture. **u**–**x** Change of drought risk, compared to present conditions: Higher temperatures increase the risk of severe drought on all sites. The KB and JU site show the highest vulnerability. On these sites, a temperature rise of 4 °C more than doubles the probability for severe drought. On the other hand, the elongation of the growing season has almost no impact on the drought risk. The driest site (EX) exhibits the weakest increase in the risk of severe drought

**Fig. 5:**
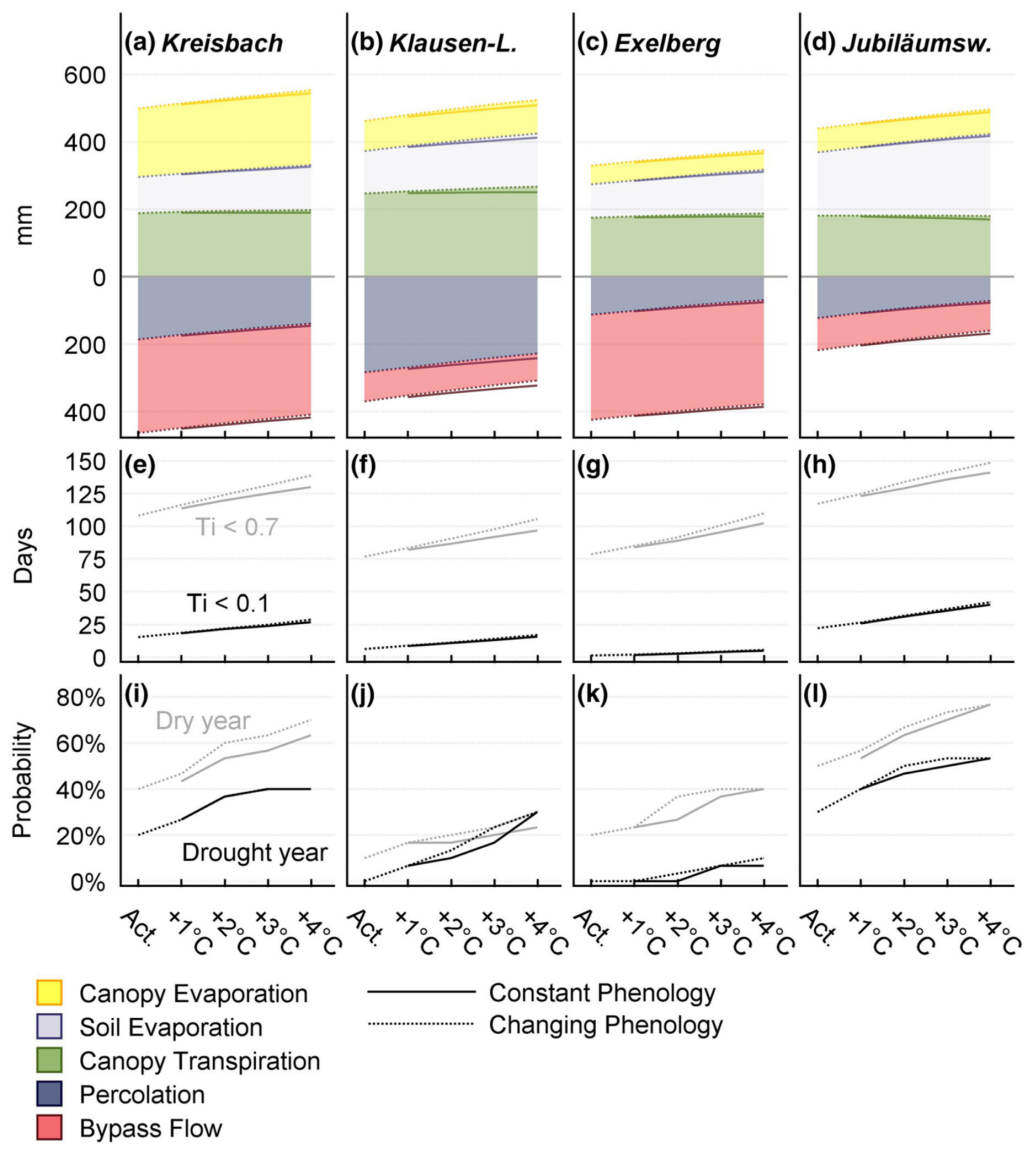
**a**–**d** The influence of rising temperatures on the stands’ annual export flux sums. Liquid and gaseous fluxes are displayed below and above zero, respectively. Warmer temperatures decrease the fraction of percolation, while (unproductive) soil evaporation rises. Although potential evapotranspiration rises, annual transpiration shows almost no response to higher temperature. The reason for this can be found in drier soils, which are favoring soil evaporation. **e**–**h** Days with water stress (defined as *T*_i_ < 0.7: grey line) and soil drought (*T*i < 0.1: black line) within the growing season (from 25th March to 11th November): All sites display a distinct increase of dry days per year, with rising temperature. Except on the EX site, higher temperatures lead also to an increased frequency of drought days per year. In both cases, the effect of elongated growing seasons is almost negligible. **i**–**l** We define dry years as years with more than 120 days of *T*_i_ < 0.7 during the growing season; drought years are defined as years with more than 30 days of *T*_i_ < 0.1 during the growing season. Under current climate the risk for drought years does not exceed 20%, meaning drought years occur roughly every 5th year. On the KL and EX site, no year fulfilled the criteria for drought years, within the reference climate period. On the EX site, 4 °C warming lead also to no noteworthy increase of drought years. Under current conditions, the KB and JU site are facing the highest risk of severe soil water deficit. Considering an exacerbation due to rising temperatures, it seems possible that these stands might undergo future changes in species composition and productivity

The simulated temperature rise had a strong effect on the stands’ water balance. Apparently, higher temperatures during the growing season led to drier soils (Fig. 4a–d). Gaseous water exports increased, while liquid exports decreased. Overall, the soil evaporation partition was affected positively, the percolation water fraction was affected negatively by warmer conditions (Fig. 5a–d). Increased ET was shifting soil moisture to lower levels, favoring the evaporation partition of ET, which is capable to deplete soil water at moisture levels below the wilting point, inducing severe drought.

Under current conditions, the KR and JU site experience frequent dry and drought years (Fig. 5i,l). Here, rising temperatures might lead to a drastic exacerbation of the situation. The reason, that the KR site (which is receiving the highest amount of precipitation) shows a similar behavior as the JU site (which is receiving the lowest annual precipitation sums), can be found in an unfavorable combination of high canopy evaporation rates with high bypass water fluxes (Table 4). At the end, only a small partition of the incoming water is available for plant consumption.

Under current conditions, drought is a rare phenomenon on the KL and EX site (Fig. 5f, g): The KL stand receives relatively high annual precipitation sums. Only a small percentage is lost by interception (Table 4). Paired with a high infiltration capacity, this has a beneficial effect on the stands’ water supply. But also here, rising temperatures led to an increase of dry and drought years. Due to the favorable current state, the impact seemed to be less pronounced. It appears paradox, that the driest site experiences the lowest risk for severe soil drought, but the EX forest benefits from a very low modeled wilting point (see Table 3). This is enabling plant water supply at low soil moisture levels (Fig. S8). On this site, severe soil drought does only occur infrequently under present conditions and also rising temperatures lead to no significant increase of the drought risk (Fig. 4 s, w).

An earlier start of the growing season let to higher *E*_C_ water fluxes from spring to early summer (Fig. 3i–l). The effect was amplified by high rates of potential evapotranspiration during this period. Early LU had an intensifying effect on soil water stress in summer. As a consequence, the scenarios which considered changes in spring phenology experienced a drastic transpiration drop in mid-summer. On the contrary to soil water stress, extended growing seasons had almost no effect on the frequency and duration of severe drought. On all sites, there was a high probability for moderate soil water deficit (*T*_i_ < 07) during the entire growing season, with a weakly pronounced maximum at the beginning of summer. On the other hand, the risk for severe drought (*T*_i_ < 0.1) shows a very distinct peak at the end of August (Fig. 4q–t).

**Fig. 6:**
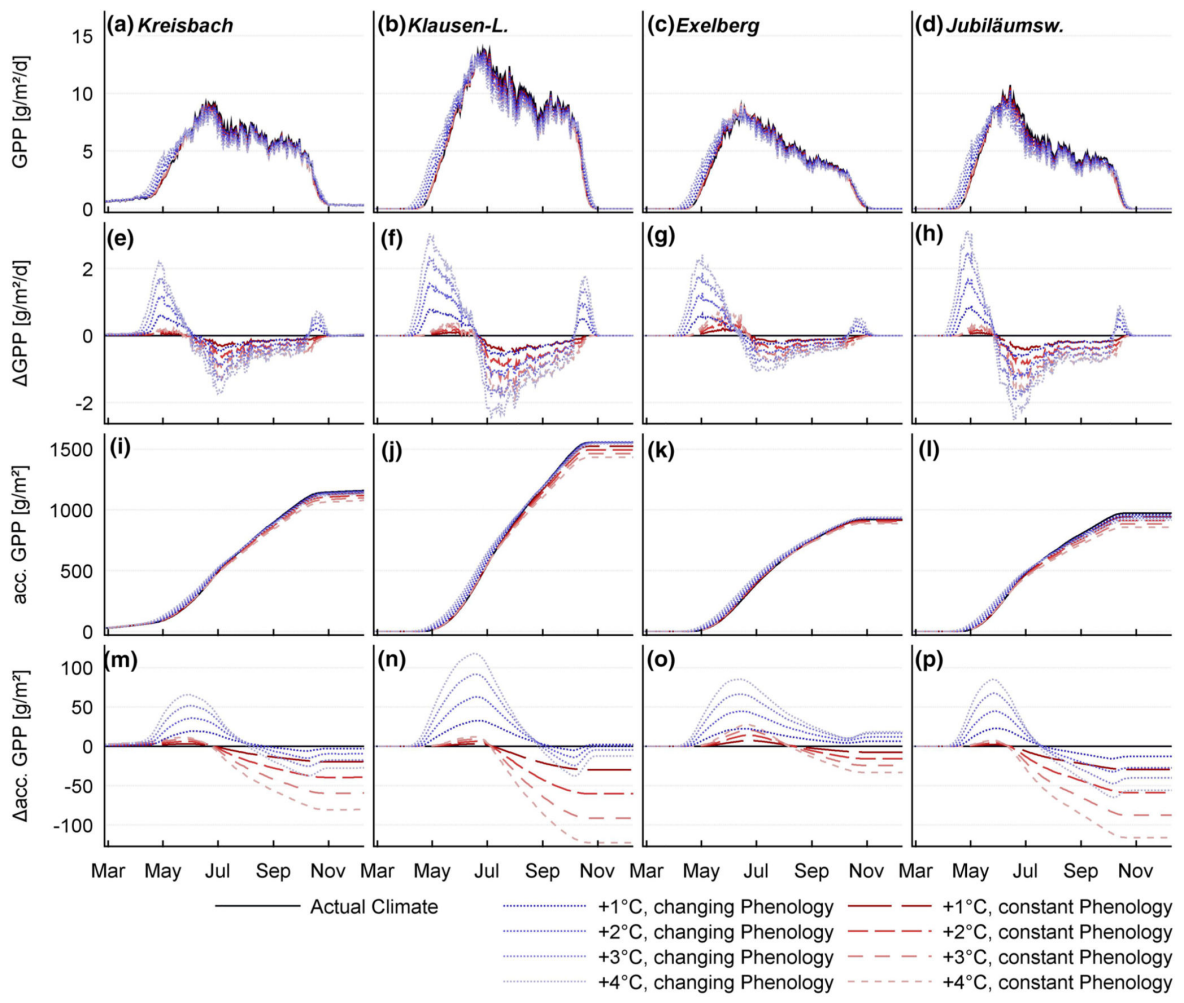
Gross primary production on a day of year base: GPP was calculated as the product of transpiration water fluxes and the estimated water use efficiency. **a**–**d** GPP shows a pattern, very similar to transpiration. **e**–**h** Change of daily GPP compared to the reference scenario: Higher temperatures in spring and earlier LU accelerate the assimilation early in the season, before soil moisture deficit hampers primary production in mid-summer. **i**–**l** Accumulated GPP over the year: analogue to annual transpiration, the KL plot **j** shows the highest productivity. **m**–**p** Change of the accumulated primary production to the reference climate period: All warming scenarios show an advance in production in late spring, which dissipates in summer. Without consideration of a change in the growing season length, all warming scenarios respond with decreased annual assimilation. Due to a compensating effect of elongated growing seasons, rising temperatures lead to no net change of the stands’ annual primal production. Again, the EX stand represents an exception. It appears paradox that the driest site seems to be also the only site, which might benefit from higher temperatures!

Longer growing seasons are assumed to widen the timeframe for potential C-assimilation (Gunderson et al. 2012; Vitasse et al. 2009). Without consideration of the growing season elongation caused by higher temperatures, all sites responded with decreased annual GPP due to water stress in mid-summer (Fig. 6e–h). In our simulation, prolonged growing seasons led to a marked increase of productivity at early stages of the growing season, followed by a significant depression from July to September (compare also Bergh et al. 2003). Growing season elongations were hardly sufficient to compensate for the productivity drop in mid-summer, caused by soil water stress. Only the EX site exhibits a net gain of GPP due to the combination of higher temperatures and longer growing seasons (Fig. 6o).

Falling in a time of high potential productivity, the extension at the start of the growing season had a stronger effect than the delay of autumn senescence (compare Gunderson et al. 2012).

## Conclusion

In this work, a simulator is presented, aiming to depict the water fluxes and the phenological dynamics of beech forest stands. Covering forest sites dominated by deciduous trees, the formulation comprises routines for the inter- and intra-annual dynamics of the vegetation cover. A routine is introduced, calculating fog precipitation as a function of air temperature, relative humidity and wind speed. Furthermore, precipitation interception is calculated, utilizing a function based on the Langmuir isotherm. Soil processes (saturation excess overflow, percolation, soil evaporation, and transpiration) are described, utilizing a zero dimensional box model approach. Despite the strong simplification of the plant–soil system, this approach was sufficient to provide an accurate prediction of the vertically integrated soil moisture on both investigated plots. The formulation, which’s set up is exhibited in this article and the supplementary, it then used to assess changes in the water balance, caused by increasing temperatures.

Climate change might affect Central European forests in multiple ways. Along with the rise in temperature, the sites will face the effects of changing precipitation patterns, rising atmospheric CO_2_ concentration, and the change of frequency, duration and intensity of extreme weather events. On the biotic side, the increased occurrence of pathogens will put even more pressure on forest ecosystems. In this work, we focused solely on the effect of rising temperatures of the stands’ water balance. The result of the simulation leads to the suggestion of mitigating measures.

To decrease the impact of drought on the forest stand, Bolte et al. (2007) point out the importance of a deliberate water resource management. They claim, that reduction in the shelterwood (1) decreases the overall water consumption of the stand and the (2) drought risk of overtopped trees. Our modeling work points out the opposite: A reduction of leaf area might lead to reduced water consumption by stand transpiration. On the other hand, unproductive soil evaporation is promoted by the increased aeration and light availability at the forest ground. At soil moisture approaching the wilting point, trees respond by actively shutting down transpiration. Only soil evaporation is capable to cause a noteworthy soil water depletion at soil moisture levels close to the wilting point, inducing severe drought. A dense canopy cover might help to counteract this, by suppressing evaporative fluxes from the soil. In that context, the strict differentiation between moderate soil water deficit and severe soil drought seems reasonable. Soil water deficit during the growing season seems to be a very common state on the investigated sites. It is clearly represented through our simulations that trees decrease their productivity during periods of water stress, but overall, they seem sufficiently adapted to such conditions. Only a strong increase in the frequency of moderate soil water stress might induce a shift in the composition towards species, with higher tolerance to soil water deficit. Severe drought on the other hand, might actively lead to severe damage of the stand (compare Barigah et al. 2013), resulting in more abrupt, or even catastrophic, changes in the appearance of the forest.

## Supplementary Material

**Electronic supplementary material** The online version of this article (https://doi.org/10.1007/s40808-019-00602-1) contains supplementary material, which is available to authorized users.

Supplementary data

Appendix

## Figures and Tables

**Table 1:** Parameterization results of the phenological module

Leaf unfolding		Leaf coloring	
DoY_0,LU_	4.353	DoY_0,LC_	108.451
*T*_0,LU_	− 10.820	DoY_1,LC_	167.207
*T*_1,LU_	30.747	DoY_2,LC_	219.214
*m*_LU_	0.362	DoY_3,LC_	286.441
*x*_LU_	2.921	*h*_LC_	− 0.618
		*k*_LC_	4.002
		*d*_LC_	261.107
*n*	65	*n*	65
NSE	0.895	NSE	0.733
RMSE	1.806	RMSE	1.736

Optimization was conducted, using a combination of simulated annealing and the Gauss–Newton algorithm. Performance criterion was the Nash–Sutcliffe model efficiency

**Table 2:** The model calibration was conducted, utilizing soil moisture records of the entire model timeframe

Site	Timeframe	*z* (cm)	*n*	NSE	RMSE
Kreisbach	04/14/1999–02/10/2003	00–50	1294	0.9262	0.0132
Klausen-Leopoldsdorf	10/01/2006–09/30/2013	00–50	2262	0.9303	0.0163
Exelberg	10/01/2009–09/30/2012	00–50	33	0.9148	0.0152
Jubiläumswarte	10/01/2009–09/30/2012	00–50	33	0.9225	0.0176

Optimization was performed, using a simulated annealing algorithm. Performance criterion was the Nash–Sutcliffe Efficiency *n* number of utilized observations, *NSE* Nash–Sutcliffe Efficiency, *RMSE* root mean squared error (LL^−1^)

**Table 3:** Parameter optimization results for all 4 investigate sites

Parameter	Description	Unit	KB	KL	EX	JU
rH_0_	Lower relative humidity threshold for fog precipitation	rH	99.99	99.99	94.34	93.93
*f*_c_	Fog coefficient		0	0	0.516	0.494
DoY_CC_	Canopy closure	DoY	221.4	222.9	173.3	180.5
*l*_LC_	Duration of leaf senescence	Days	22.88	20.94	25.14	13.60
ID_LAI_	Indeciduous fraction of leaf area at canopy closure		0.079	0	0	0
*C*_max,LAI0_	Canopy interception capacity at Kc_LAI_ = 0	mm	2.221	0.632	0.325	0.309
*C*_max,LAI1_	Canopy interception capacity at Kc_LAI_ = 1	mm	4.326	2.701	0.678	1.230
*K*_i,LAI0_	Interception function shape parameter at Kc_LAI_ = 0		4.354	3.777	2.665	2.832
*K*_i,LAI1_	Interception function shape parameter at Kc_LAI_ = 1		5.342	6.712	4.678	5.339
Kc_canopy_	Crop coefficient for canopy evaporation		1.102	0.472	0.787	0.822
*τ*_LAI0_	Maximum radiation transmittance coefficient at Kc_LAI_ = 0		0.742	0.701	0.802	0.805
*τ*_LAI1_	Minimum radiation transmittance coefficient at Kc_LAI_ = 1		0.390	0.321	0.319	0.291
*T*_snow_	Upper threshold temperature for snowfall	°C	1.388	0.203	1.706	1.509
*T*_melt_	Lower threshold temperature for snowmelt	°C	1.972	1.508	1.897	0.938
DDF_melt_	Degree day factor for snowmelt	mm °C^−1^ day^−1^	2.165	2.982	0.813	0.686
*f*_by_	Water fraction, bypassing the soil box		0.362	0.083	0.437	0.112
*k*_sat_	Saturated conductivity	LL^−1^ day^−1^	0.071	0.038	0.017	0.021
*θ*_m_	Shape parameter for percolation response		0.528	0.728	0.753	0.748
*θ*_sat_	Saturated water content	LL^−1^	0.502	0.520	0.369	0.420
*θ*_fc_	Field capacity	LL^−1^	0.344	0.392	0.285	0.362
θ*	Upper threshold water content for evapotranspiration limit.	LL^−1^	0.344	0.410	0.270	0.348
*θ*_pwp_	Permanent wilting point, lower threshold for transpiration	LL^−1^	0.189	0.228	0.085	0.155
*θ*_res_	Residual water content, lower threshold for soil evaporation	LL^−1^	0.008	0.003	0.012	0.003
ET_m_	Shape parameter for evapotranspiration response		0.488	0.391	0.422	0.424
Kc_tree_	Crop coefficient for vegetation at canopy closure		1.361	1.783	0.526	0.857
Kc_ground_	Crop coefficient for soil evaporation		0.364	0.517	0.209	0.398

**Table 4:** Breakdown of modeled export fluxes, given as mean annual sums and in percent of the observed precipitation

Site	Kreisbach		Klausen-Leo.		Exelberg		Jubiläumswarte	
*n* (years)	30		30		30		30	
Unit	mm a^−1^	%	mm a^−1^	%	mm a^−1^	%	mm a^−1^	%
Precipitation (+ fog)	961.0	100	831.1	100	751.5	100	654.5	100
Fog	0	0	0	0	6.0	0.8	6.8	1.1
Canopy evaporation	202.8	21.7	88.4	10.8	54.4	7.4	68.4	10.7
Infiltration	480.8	49.7	656.4	79.1	386.1	51.5	492.4	75.7
Soil evaporation	106.6	11.6	127.1	15.6	100.1	13.8	189.0	30.1
Transpiration	189.1	19.8	246.3	29.8	174.4	23.5	180.9	27.6
Bypass flow	277.6	28.6	85.5	10	311.1	41.4	93.9	13.9
Percolation	185.1	18.4	283.0	33.7	111.8	14.6	122.9	18.6
