# Supplementary material for: The impact of rising temperatures on water balance and phenology of European beech (Fagus sylvatica L.) stands
Source: Model Earth Syst Environ. Author manuscript; Available in PMC 2019 Dec 1. (PMC6814441; doi:10.1007/s40808-019-00602-1)
Supplement: Supplementary data [file EMS84601-supplement-Supplementary_data.docx]

**The Impact of rising Temperatures on Water Balance and Phenology of European Beech (*Fagus Sylvatica* L.) Stands.**

**Supplementary Information**

Klaus Dolschak^a,*^, Karl Gartner^b^ & Torsten W. Berger^a^

^a^Department of Forest- and Soil Sciences, Institute of Forest Ecology,
University of Natural Resources and Life Sciences (BOKU), Peter Jordan-Straße 82, 1190 Vienna, Austria

^b^Federal Research and Training Centre for Forests, Natural Hazards and Landscape, Department of Forest Ecology and Soil, Seckendorff-Gudent-Weg 8, 1131 Vienna, Austria

^*^corresponding author: Tel: +43-6507951941

Email: [klaus.dolschak@gmx.at](mailto:klaus.dolschak@gmx.at)

**Fig. S1** Flow diagram of the water balance model. The water input to the system consists of observed precipitation and sim­ulated fog interception. Solid lines, dashed lines and dotted lines reflect liquid, solid and gaseous water fluxes, respectively. Rounded boxes represent pools which are described, in form of differential equations. Aboveground processes are calculated sequential. Belowground processes (bypass flow, soil eva­poration, transpiration, and percolation) are handled parallel.

**
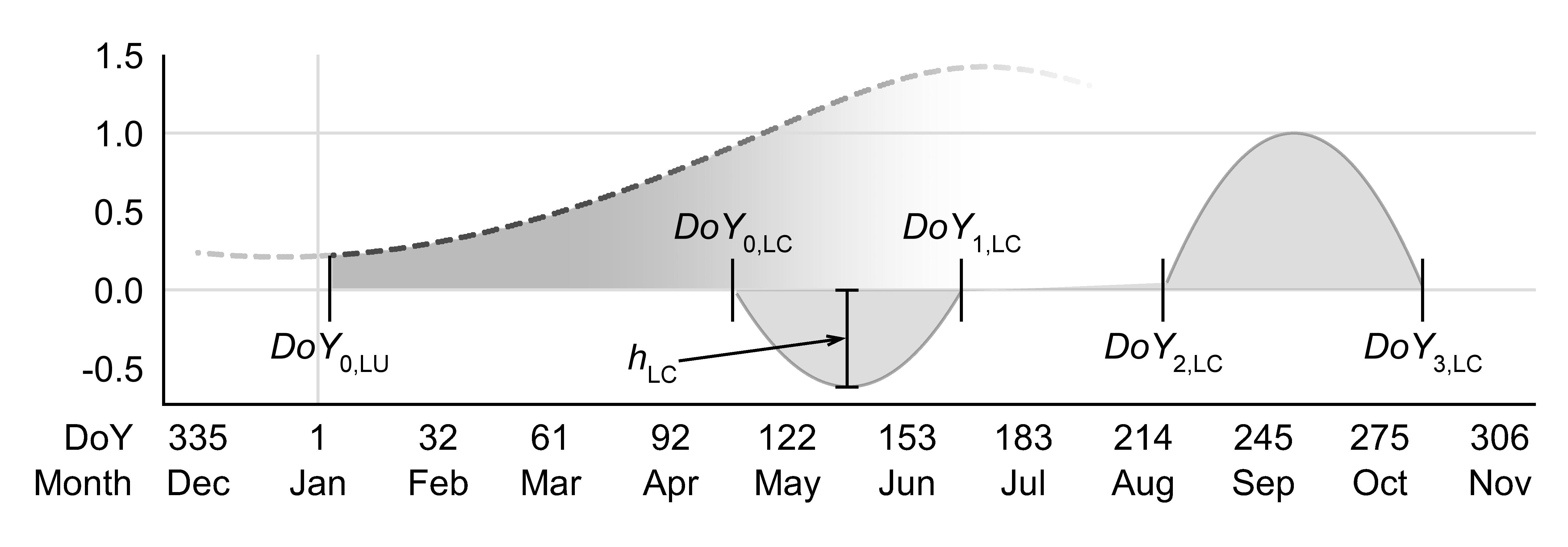
Fig. S2** Air temperature multi­pliers, utilized in the assessment of LU and LC. The factor *f*_dl_ (dashed line), calculated as func­tion of day-length, serves in the determination of the onset of the growing season. *DoY*_0,LC_ marks the potential start of the temp­erature accumulation in winter. For the calculation of the timing of LC, *T*_mean_ is weighted by a parabolic shaped function, within 2 intervals. Assuming, a negative influence of high spring temperatures on the timing on senescence, the parabolic function in the first interval takes negative values. Due to a positive effect of high late summer on growing season length, the multiplier of the second interval is also positive. Utilizing the weighted average of both temporal windows combined, the timing of LC is calculated in a simple linear model.

**SI 1. Seasonal leaf area development**

In this section a function is presented, describing relative leaf area (*Kc*_LAI_) in an interval from 0 to1 (see Fig. S3). Five phenological stages are distinguished: (1) Dormancy, (2) a linear onset of the growing season, (3) a polynomic convergence with the maximum, (4) a plateau stage, and (5) senescence, which is described, using a third order polynomial ([smoothstep function, compare Dolschak et al. 2015](#_ENREF_4)). Five parameters define the shape of the function. Four key *DoYs* (*DoY*_LU_: Leaf unfolding, *DoY*_CC_: Canopy closure, *DoY*_LC_: Leaf coloring, *DoY*_LE_: Litterfall end) define the seasonal development; the parameter *ID*_LAI_ accounts for a relative fraction of indeciduous trees.

|  | ${Kc}_{\mathrm{LAI}}={ID}_{\mathrm{LAI}}+\left( 1-{ID}_{\mathrm{LAI}} \right)\left\{ \begin{matrix} 0 & DoY\leq{DoY}_{\mathrm{LU}} \vee DoY>{DoY}_{\mathrm{LE}} \\ \frac{4t_{\mathrm{LU}}}{3} & {DoY}_{\mathrm{LU}}< DoY\leq{DoY}_{2/3} \\ \frac{8t_{\mathrm{LU}}-4{t_{\mathrm{LU}}}^{2}-1}{3} & {DoY}_{2/3}< DoY\leq{DoY}_{\mathrm{CC}} \\ 1 & {DoY}_{\mathrm{CC}}< DoY\leq{DoY}_{\mathrm{LC}} \\ 1-{3t_{\mathrm{LC}}}^{2}+2{t_{\mathrm{LC}}}^{3} & {DoY}_{\mathrm{LC}}< DoY\leq{DoY}_{\mathrm{LE}} \end{matrix} \right.$ | (S) |
| --- | --- | --- |

The temporal center of the unfolding period marks the transition from linear increment to polynomic flattening.

|  | ${DoY}_{2/3}=\frac{{DoY}_{\mathrm{LU}}+{DoY}_{\mathrm{CC}}}{2}$ | (S) |
| --- | --- | --- |

The end of the growing season calculates as the sum of the mean start of senescence and the length of the litterfall event.

|  | ${DoY}_{\mathrm{LE}}={DoY}_{\mathrm{LC}}+l_{\mathrm{LC}}$ | (S) |
| --- | --- | --- |

The *DoY* is normalized inside the corresponding interval by following equations:

|  | $t_{\mathrm{LU}}=\frac{DoY-{DoY}_{\mathrm{LU}}}{{DoY}_{\mathrm{CC}}-{DoY}_{\mathrm{LU}}}$ | (S) |
| --- | --- | --- |
|  | $t_{\mathrm{LC}}=\frac{DoY-{DoY}_{\mathrm{LC}}}{l_{\mathrm{LC}}}$ | (S) |

**
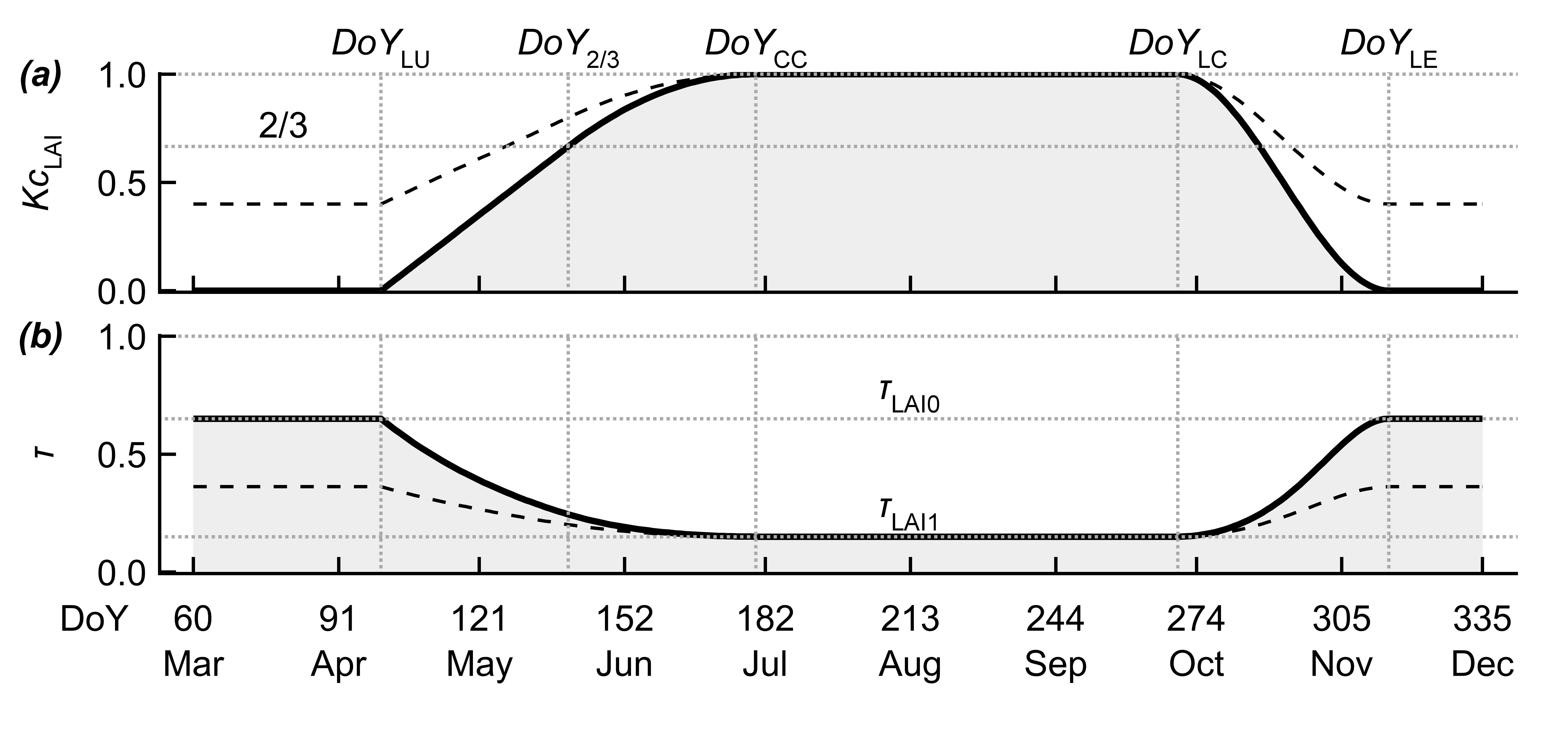
**

**Fig. S3** (a) Construction of rel­ative LAI index (*Kc*_LAI_) with para­meters *DoY*_LU_ =100, *DoY*_CC_=180, *DoY*_LC_=270, *DoY*_LE_=315. The first half of the leaf unfolding period (*DoY*_LU_- *DoY*_2/3_) is described utilizing a linear equation. The second half (*DoY*_2/3_- *DoY*_CC_) is described using a second order polynomial. Leaf senescence (*DoY*_LC_- *DoY*_LE_) is modeled with the smoothstep function. Solid and the dashed line account for an indeciduous fraction (*IC*_LAI_) of 0% and 40%, respectively. (b) Definition of the relative radiation transmission (τ) as a function of *Kc*_LAI_ using τ_LAI0_=0.65 for the dormant season and τ_LAI1_=0.15 at full vegetation cover. The solid and the dashed line again account for an evergreen fraction of 0% and 40%, respectively.

**SI 2. Fog interception precipitation**

This very simple static model employs 3 parameters. Input variables are *rH* (daily average), daily mean temperature and wind speed. The mean temperature is used to calculate saturation vapor pressure (*E*_sat,_ kPa), and subsequently the saturation water content of the air (*ρ*, kg m^-3^).

Having available only observations of daily mean *rH*, we lack direct information on the temporal frequency within one day, saturation vapor pressure is actually reached, and thus conditions might be suitable for the generation of fog precipitation. Therefore, we introduce the dimensionless model variable *f*_sat_, which enables conversion of *rH* into an estimate of the relative frequency of saturation events during one day. The calculation utilizes the smoothstep function. It assumes values in a range from 0 to 1. The parameter (*rH*_0_) defines a supposed minimum of the daily average *rH*, where fog is occurring. At *rH* =100, fog is supposed to occur the entire day. The setting up of this function is described in the Appendix (Eq. A1-A3)

|  | $f_{\mathrm{sat}}=smooth(rH,{rH}_{0},100,0,1)$ | (S) |
| --- | --- | --- |

As a simple analytical model, fog drip can be described as the product of liquid water content (*LWC*, kg m^-3^) and deposition velocity of aerosol particles ([Slinn 1982](#_ENREF_11)), where deposition velocity is assumed to be almost proportional to the wind speed above the canopy. Due to unavailability of *LWC* measurement, proportionality of *f*_sat_ and the mean daily liquid water content is assumed.

|  | $f_{\mathrm{sat}}\sim LWC$ | (S) |
| --- | --- | --- |

Therefore, the resulting model can be written as the product of the relative frequency of daily saturation events, the water content at saturation, the mean wind speed (*u*_2_, m s^-1^), and a coefficient *f*_c_, which scales this product to actual daily fog precipitation sums [mm].

|  | $P_{\mathrm{fog}}=f_{\mathrm{sat}}f_{c}u_{2}$ | (S) |
| --- | --- | --- |

The fog precipitation module is very sensitive to humidity data. Minor differences in the measurement of relative humidity will yield highly different results in the amount of modeled fog precipitation. If no data for validation is available, and accordingly the data quality of humidity measurement in the range close to saturation water content is poor, the impact of whole module can be reduced by setting *f*_c_ to a low value, or even zero. The sum of observed precipitation and fog precipitation state the input at the canopy.

|  | $P_{\mathrm{sum}}=P_{\mathrm{obs}}+P_{\mathrm{fog}}$ | (S) |
| --- | --- | --- |

**SI 3. Precipitation Interception**

In this work, we present an analytical precipitation interception module, which can be interpreted as an analogue to the Langmuir adsorption isotherm, where interception (*I*) and throughfall (*Tf*) are replacing occupied sorption sites and solution concentration, respectively. *K*_i_ corresponds to the Langmuir adsorption constant. The latter defines the impermeability of the canopy at low precipitation intensity. *C*_max_ [mm] sets the maximum possible charge of the canopy.

|  | $I=\frac{K_{i}C_{\max}Tf}{1+K_{i}Tf}$ | (S) |
| --- | --- | --- |

The solving of the equation for *Tf*, is described in the Appendix (Eq. A8 – A11). The determination of throughfall utilizes the sum of observed precipitation and modeled fog precipitation, the shape parameters of the Langmuir type equation, plus 2 auxiliary variables (*Tf*_h_, *P*_h_) which are derived, from the canopy storage of the previous day (*C_t_*_-1_).

|  | ${Tf}_{h}=\frac{C_{t-1}}{K_{i}\left( C_{\max}-C_{t-1} \right)}$ | (S) |
| --- | --- | --- |
|  | $P_{h}={Tf}_{h}+C_{t-1}$ | (S) |

Actual throughfall is determined.

|  | $Tf=tru\left( P_{\mathrm{sum}}+P_{h},C_{\max},K_{i} \right)-{Tf}_{h}$ | (S) |
| --- | --- | --- |

Actual interception is calculated as the difference of incoming precipitation and throughfall:

|  | $I=P_{\mathrm{sum}}-Tf$ | (S) |
| --- | --- | --- |

Canopy storage prior to canopy evaporation is defined as:

|  | $C_{\mathrm{init}}=C_{t-1}+I$ | (S) |
| --- | --- | --- |

The determination of the canopy interception evaporation (*E*_I_) utilizes the FAO-Penman Monteith reference evapotranspiration (*ET*_o_) ([Allen et al. 1998](#_ENREF_1)), modified with an empirical coefficient (*Kc*_canopy_).

|  | $E_{I}=\left\{ \begin{matrix} {ET}_{o}{Kc}_{\mathrm{canopy}}, & C_{\mathrm{init}}\geq{ET}_{o}{Kc}_{\mathrm{canopy}} \\ C_{\mathrm{init}}, & C_{\mathrm{init}}<{ET}_{o}{Kc}_{\mathrm{canopy}} \end{matrix} \right.$ | (S) |
| --- | --- | --- |

At last, the actual canopy storage is calculated.

|  | $C_{t}=C_{\mathrm{init}}-E_{I}$ | (S) |
| --- | --- | --- |

As our model focuses on deciduous forest stands, it seems indispensable to consider a change of interception characteristics with the change of canopy cover throughout the year. Analogue to [Bastiaanssen et al. (2012)](#_ENREF_2) and [van Dijk and Bruijnzeel (2001)](#_ENREF_12), a linear relationship of leaf area index and canopy storage capacity is assumed. This is implemented by defining 2 parameters: (1) a minimum value at *Kc*_LAI_=0, is reflecting winter conditions with only stems and bare branches intercepting precipitation, and (2) a maximum value at full vegetation cover (*Kc*_LAI_=1). For transition periods in between minimum and maximum a linear equation is employed, using both parameters.

|  | $C_{\max}={Kc}_{\mathrm{LAI}}\left( C_{max,LAI1}-C_{max,LAI0} \right)+C_{max,LAI0}$ | (S) |
| --- | --- | --- |

Besides a change of *C*_max_, we hypothesize also a change in the permeability of the canopy throughout the season. In this work, the permeability of the canopy is controlled by the parameter *K*_i_. Analogue to canopy storage capacity, it is assumed that a dense vegetation cover is leading to low permeability. The dependency of this shape parameter on leaf area is set analogue to the dependency of canopy storage capacity.

|  | $K_{i}={Kc}_{\mathrm{LAI}}\left( K_{i,LAI1}-K_{i,LAI0} \right)+K_{i,LAI0}$ | (S) |
| --- | --- | --- |

**
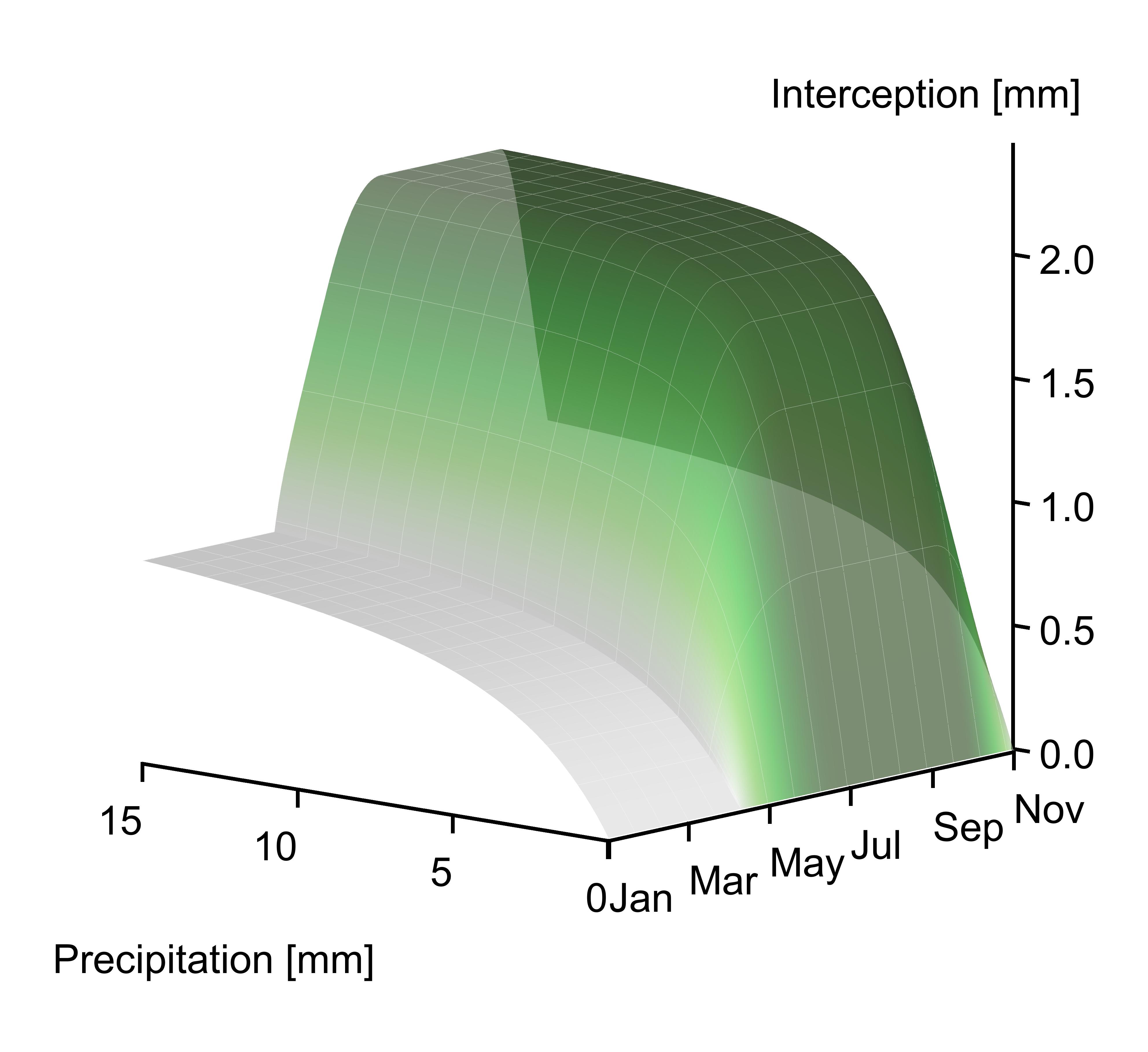
Fig. S4** Seasonal course of precipitation dependent interception. For the parameters, used in the construction of *Kc*_LAI_ see Fig. S3. Utilized interception parameters were: *C*_max,LAI0_=1 mm , *C*_max,LAI1_=2.2 mm, *K*_i,LAI0_=0.3, *K*_i,LAI1_=4. A high *K*_i_ value is leading to low permeability of the canopy at low precipitation sums. Note that the displayed response surface only applies to canopies, which were dry prior to the precipitation event!

**SI 4. Snow dynamics**

In the presented work, potential snowfall and snowmelt are processes which depend on daily temperature. In both cases, a critical temperature is defined, which allows the decision whether incoming precipitation falls as snow or rain, as well as snowmelt is taking place. Temperature threshold models might have shortcomings when working with mean temperatures on a daily basis. In this case it is possible that the actual temperature crosses the threshold during the day, resulting in a transition of the state (rain/snow, frost/melt), undetected by the model ([Hock 2003](#_ENREF_7)). One possibility to tackle this problem would be the replacement of the threshold with a transition interval ([Feiccabrino and Lundberg 2008](#_ENREF_6)), or more resource demanding, the utilization of temperature data with a higher temporal resolution to cover temperature fluctuations within the day ([Hock 2003](#_ENREF_7)). There are possibilities to circumvent this necessity. For calculating relative partitions of snow and rain precipitation, [Willen et al. (1971)](#_ENREF_13) first presented an approach, using observed *T*_min_ and *T*_max_ and a critical temperature (*T*_snow_).

|  | $p_{\mathrm{snow}}=\left\{ \begin{matrix} 0, & T_{\mathrm{snow}}\leq T_{\min} \\ \frac{T_{\mathrm{snow}}-T_{\min}}{T_{\max}-T_{\min}}, & T_{\min}<T_{\mathrm{snow}}<T_{\max} \\ 1, & T_{\mathrm{snow}}\geq T_{\max} \end{matrix} \right.$ | (S) |
| --- | --- | --- |

This way the probability of a complete misjudgment of the precipitation phase is reduced ([Federer 1995](#_ENREF_5)). Actual rainfall and snowfall are then calculated as:

|  | $P_{\mathrm{rain}}=Tf\left( {1-p}_{\mathrm{snow}} \right)$ | (S) |
| --- | --- | --- |

|  | $P_{\mathrm{snow}}=Tfp_{\mathrm{snow}}$ | (S) |
| --- | --- | --- |

In this work, the liquid water equivalent (LWE, mm) is the unit, used in all snow calculations. The determination of potential snowmelt is based on the classical degree-day method ([Rango and Martinec 1995](#_ENREF_10)), where snowmelt is assumed to occur, when the actual temperature exceeds a threshold (*T*_melt_). The amount is assumed proportional to the difference of both temperatures. Dealing with *T*_min_ and *T*_max_, the approach is modified. The variable *f*_melt_ can be described as temporal fraction of a day, the critical temperature for snowmelt is exceeded.

|  | $f_{\mathrm{melt}}=\left\{ \begin{matrix} 1, & T_{\mathrm{melt}}\leq T_{\min} \\ \frac{T_{\max}-T_{\mathrm{melt}}}{T_{\max}-T_{\min}}, & T_{\min}<T_{\mathrm{melt}}<T_{\max} \\ 0, & T_{\mathrm{melt}}\geq T_{\max} \end{matrix} \right.$ | (S) |
| --- | --- | --- |

The mean temperature for the temporal fraction above the threshold (*m*_melt_) is calculated.

|  | $m_{\mathrm{melt}}=\left\{ \begin{matrix} \frac{T_{\max}+T_{\min}}{2}, & T_{\mathrm{melt}}\leq T_{\min} \\ \frac{T_{\max}+T_{\mathrm{melt}}}{2}, & T_{\min}<T_{\mathrm{melt}}<T_{\max} \end{matrix} \right.$ | (S) |
| --- | --- | --- |

Potential snowmelt (*pot*_melt_) is then described, as product of the temporal fraction above the threshold, the temperature difference, and an empirical degree-day factor (*DDF*_melt_, mm°C^-1^d^-1^).

|  | ${pot}_{\mathrm{melt}}=f_{\mathrm{melt}}\left( m_{\mathrm{melt}}-T_{\mathrm{melt}} \right){DDF}_{\mathrm{melt}}$ | (S) |
| --- | --- | --- |

The snowpack storage (mm liquid water equivalent), prior to snowmelt is calculated:

|  | ${SP}_{\mathrm{init}}={SP}_{t-1}+P_{\mathrm{snow}}$ | (S) |
| --- | --- | --- |

Actual snowmelt is defined.

|  | $P_{\mathrm{melt}}=\left\{ \begin{matrix} {pot}_{\mathrm{melt}}, & {SP}_{\mathrm{init}}\geq{pot}_{\mathrm{melt}} \\ {SP}_{\mathrm{init}}, & {SP}_{\mathrm{init}}<{pot}_{\mathrm{melt}} \end{matrix} \right.$ | (S) |
| --- | --- | --- |

Finally, the actual snowpack storage is determined.

|  | ${SP}_{t}={SP}_{\mathrm{init}}-P_{\mathrm{melt}}$ | (S) |
| --- | --- | --- |

**SI5. Soil moisture dynamics**

At the soil surface, the pathway for incoming water is branching up into bypass flow and soil infiltration. In this work, bypass flow is defined as the fraction of incoming water which hits the forest ground surface, but is not coming in closer contact with the soil matrix. In our formulation, bypass flow is generated 2 ways. The first bypass partition (*B*_1_) is calculated by multiplying the sum of rainfall and snowmelt with a constant factor (*f*_BY_) taking values from 0 to 1.

|  | $B_{1}=f_{\mathrm{BY}}\left( P_{\mathrm{rain}}+P_{\mathrm{melt}} \right)$ | (S) |
| --- | --- | --- |

The remaining water states the available input (*A*) to the soil box.

|  | $A=\left( P_{\mathrm{rain}}+P_{\mathrm{melt}} \right)\left( 1-f_{\mathrm{BY}} \right)$ | (S) |
| --- | --- | --- |

In this work, a zero dimensional box model approach for modeling soil moisture dynamics is presented, meaning the formulation lacks vertical or horizontal resolution within the soil column. Therefore, the soil could be considered as a box with a basal area of 1 m^2^ and a chosen soil depth (*z*_r_, mm), with uniform characteristics throughout the entire profile. The calculated soil moisture aims to resemble the observed mean water content [LL^-1^]; calculated input/output fluxes equal sums [Lm^-2^], vertically integrated over *z*_r_*.* So called box models have been reviewed and tested for their applicability in works by [Baudena et al. (2012)](#_ENREF_3) and [Kumagai et al. (2009)](#_ENREF_8).

The net soil water balance can be reduced to an ordinary differential equation were *z*_r_ resembles the active soil depth [mm], and *θ* the volumetric water content [LL^-1^].

|  | $z_{r}\frac{d\theta}{dt}=\left( A-B_{2} \right)-(E_{C}+E_{S}+P_{S})$ | (S) |
| --- | --- | --- |

The change of soil water storage can therefore be described as the difference of infiltrating water (available water minus saturation excess overflow) and the sum of transpiration, soil evaporation, and percolation. The presented formulation neglects the possibility of capillary rise, lateral flow and moisture diffusion.

While the aboveground module handles processes in a sequential way, in the soil module several nonlinear processes, which both influence and depend on the soil water content, occur simultaneously. To linearize this set of nonlinear equations the classical 4^th^ order Runge-Kutta method was implemented.

**Saturation excess overflow (*B*_2_)**

When the entire pore spaced is filled with water, the excess water (forming the second bypass partition) is calculated as:

|  | $B_{2}=\left\{ \begin{matrix} z_{r}\theta+A- z_{r}\theta_{\mathrm{sat}}, & \theta+A/{z_{r}}\geq\theta_{\mathrm{sat}} \\ 0, & \theta+A/{z_{r}}<\theta_{\mathrm{sat}} \end{matrix} \right.$ | (S) |
| --- | --- | --- |

**Evapotranspiration (ET)**

In the presented approach, the FAO-Penman-Monteith equation ([Allen et al. 1998](#_ENREF_1)) serves as the centerpiece for the determination of actual evapotranspiration rates. After calculation of the reference evapotranspiration (ET_O_), using daily averages of radiation, humidity, wind speed and temperature, it is scaled to the actual evaporation, e.g. transpiration rates, using several coefficients. Possible energy inputs by ground flux are neglected. In this work, no detailed description of the calculation of ET_O_ is given. The following section should just highlight the parts where the applied procedure differs from the standard procedures of the computation of the reference ET.

**Canopy transpiration (*E*_C_)**

Three coefficients influence the response of transpiration to the forest stand. (1) With all other controlling factors kept constant, proportionality of the relative leaf area (*Kc*_LAI_) to *E*_C_ is assumed. (2) Further, a site specific crop coefficient (*Kc*_tree_) for the selected forest stand is introduced. (3) A soil moisture dependent stress coefficient (*Kc*_s,tree_) defines the response of *E*_C_ to soil drought.

|  | ${Kc}_{s,tree}=msmooth(\theta,\theta_{\mathrm{pwp}},\theta^{*},0,1,{ET}_{m})$ | (S) |
| --- | --- | --- |

Above a critical soil moisture content *(θ***),* transpiration is assumed unlimited, with stomata fully opened. In this case model transpiration is defined solely as the product of ET_O_ the Crop Coefficient for the stand and the relative stage of the growing season. Undershooting *θ** plants begin to respond to reduced water availability by closing stomata. At soil moisture levels below the wilting point (*θ*_pwp_), all stomata are assumed fully closed, resulting in a complete shut-down of transpiration. Consistent with this behavior, a relative stress coefficient is introduced, which declines in between *θ^*^* and *θ*_pwp_ from one to zero. Many authors describe this transition using a linear relationship ([Laio et al. 2001](#_ENREF_9)). In this work, the modified smoothstep function is applied, with the shape parameter *ET*_m_. Its derivation is defined in the appendix (Equation A1, A4, A5). Actual canopy transpiration (*E*_C_) is then calculated as the product of the reference evapotranspiration and 3 coefficients.

|  | $E_{C}={ET}_{o}{Kc}_{\mathrm{tree}}{Kc}_{\mathrm{LAI}}{Kc}_{s,tree}$ | (S) |
| --- | --- | --- |

**Soil evaporation (*E*_S_)**

The calculation of *E*_S_ differs slightly from the calculation of *E*_C_: For determination of reference evapotranspiration below the plant cover (*ET*_soil_*)* global radiation below the canopy *(R*_soil_*)* is used. Therefore, the relative radiation transmittance (*τ*) is estimated as function of the actual state of vegetation cover, using Beer-Lamberts law.

|  | $\tau=\tau_{\mathrm{LAI}0}exp(ln\frac{\tau_{\mathrm{LAI}1}}{\tau_{\mathrm{LAI}0}}{Kc}_{\mathrm{LAI}})$ | (S) |
| --- | --- | --- |

*τ* _LAI1_ and *τ* _LAI0_ define transmittance at vegetation period extremes *Kc*_LAI_ *=* 1 and *Kc*_LAI_ *=*0, respectively*.* Global radiation below the canopy is calculated as product of the transmittance and the net radiation above the canopy.

|  | $R_{\mathrm{soil}}={\tau R}_{n}$ | (S) |
| --- | --- | --- |

The calculations of the stress coefficients for evaporation (*Kc*_s,soil_) and transpiration share 2 of 3 parameters. In the determination of E, *θ*_pwp_ is replaced with the residual water content (*θ*_res_), which is assumed to lie below the wilting point.

|  | ${Kc}_{s,soil}=msmooth(\theta,\theta_{\mathrm{res}},\theta^{*},0,1,{ET}_{m})$ | (S) |
| --- | --- | --- |

In the absence of a snow cover, soil evaporation is defined as the product of *ET*_soil_, the stress coefficient, and a crop coefficient for soil evaporation *(Kc*_soil_*)*. A present snowpack is assumed to suppress *E*_S_ completely.

|  | $E_{S}=\left\{ \begin{matrix} {ET}_{\mathrm{soil}}{{Kc}_{s,soil}Kc}_{\mathrm{soil}}, & {SP}_{t}=0 \\ 0, & {SP}_{t}>0 \end{matrix} \right.$ | (S) |
| --- | --- | --- |
|  |  |  |

**Percolation (*P*_s_)**

Comparable to evapotranspiration, we describe drainage as a threshold process. Below water holding capacity *(θ*_fc_*)*, gravitational potential is assumed to be fully compensated by the soil matrix potential, resulting in no change in soil water storage due to leakage. Exceeding this critical value, percolation is initiated, reaching its maximum rate *(k*_sat_*)* at soil water saturation *(θ*_sat_*)*. For modeling the transition of leakage losses between *θ*_fc_ and *θ*_sat_, ([Laio et al. 2001](#_ENREF_9)) use an exponential relationship.

|  | $P_{S}=k_{\mathrm{sat}}\frac{e^{\beta\left( \theta-\theta_{\mathrm{fc}} \right)}-1}{e^{\beta\left( \theta_{\mathrm{sat}}-\theta_{\mathbf{fc}} \right)}-1}$ | (S) |
| --- | --- | --- |

The parameter *β* defines the shape of the transition from *θ*_fc_ to *θ*_sat_. Also in this work percolation is formulated in exponential form. The parameter *θ*_m_ marks the relative position between *θ*_fc_ and *θ*_sat_ where the conductivity reaches 50% of *k*_sat_.

|  | $P_{S}=z_{r}k_{\mathrm{sat}}\left\{ \begin{matrix} 0, & \theta\leq\theta_{\mathrm{fc}} \\ \left( \frac{\theta-\theta_{\mathrm{fc}}}{\theta_{\mathrm{sat}}-\theta_{\mathrm{fc}}} \right)^{\frac{\ln0.5}{\ln\theta_{m}}}, & \theta_{\mathrm{fc}}<\theta<\theta_{\mathrm{sat}} \\ 1, & \theta\geq\theta_{\mathrm{sat}} \end{matrix} \right.$ | (S) |
| --- | --- | --- |

Finally, the stream discharge can be determined as the sum of 2 bypass fractions and percolation.

|  | $D=B_{1}+B_{2}+P_{S}$ | (S) |
| --- | --- | --- |

**
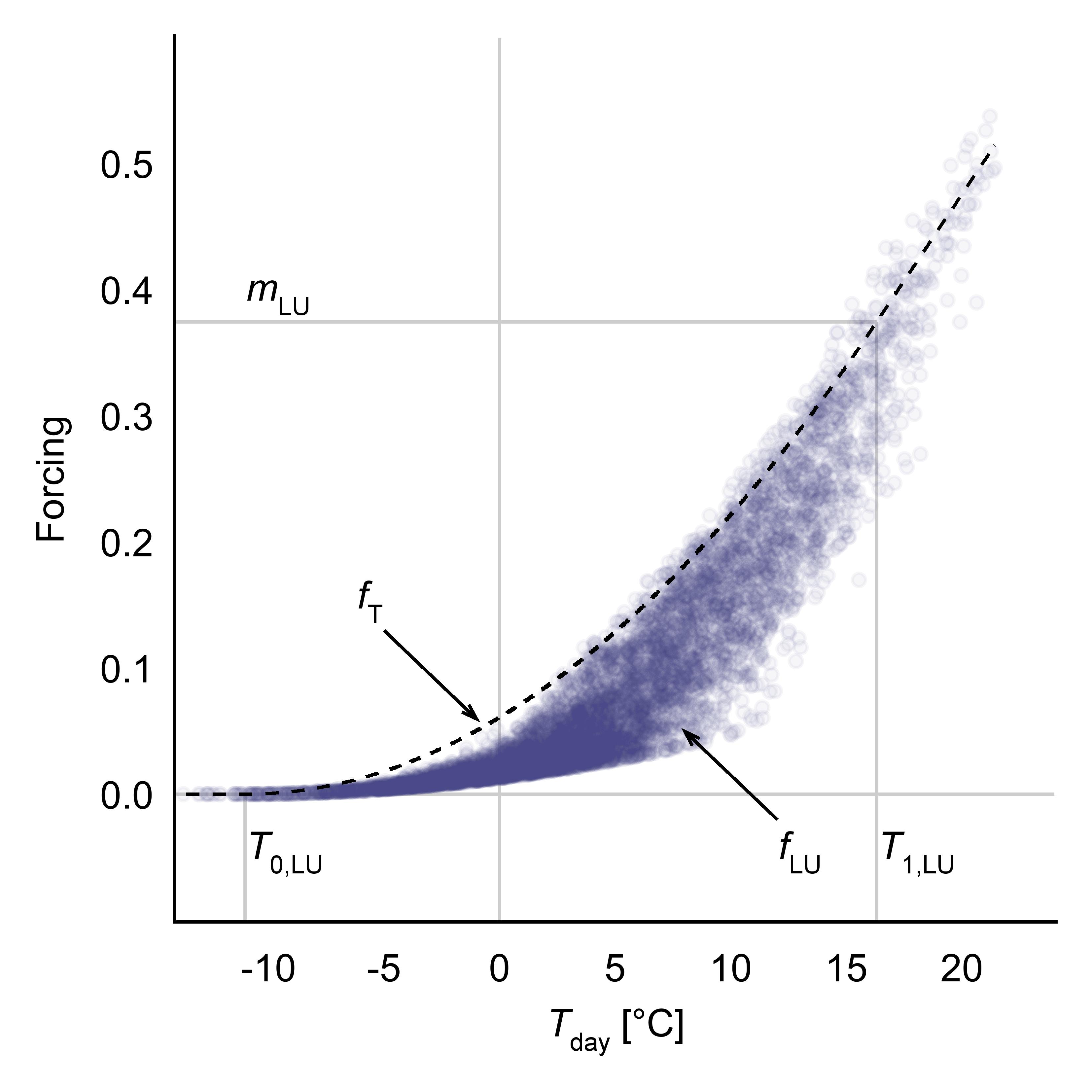
Fig. S5** Modeled temperature driven forcing (*f*_T_: dashed line) and the daily actual forcing (*f*_LU_: point cloud) in relation to daytime temperature. The onset of *f*_T_ at very low temperatures, is partially nullified by the photoperiod term, taking low values early in the year.

**
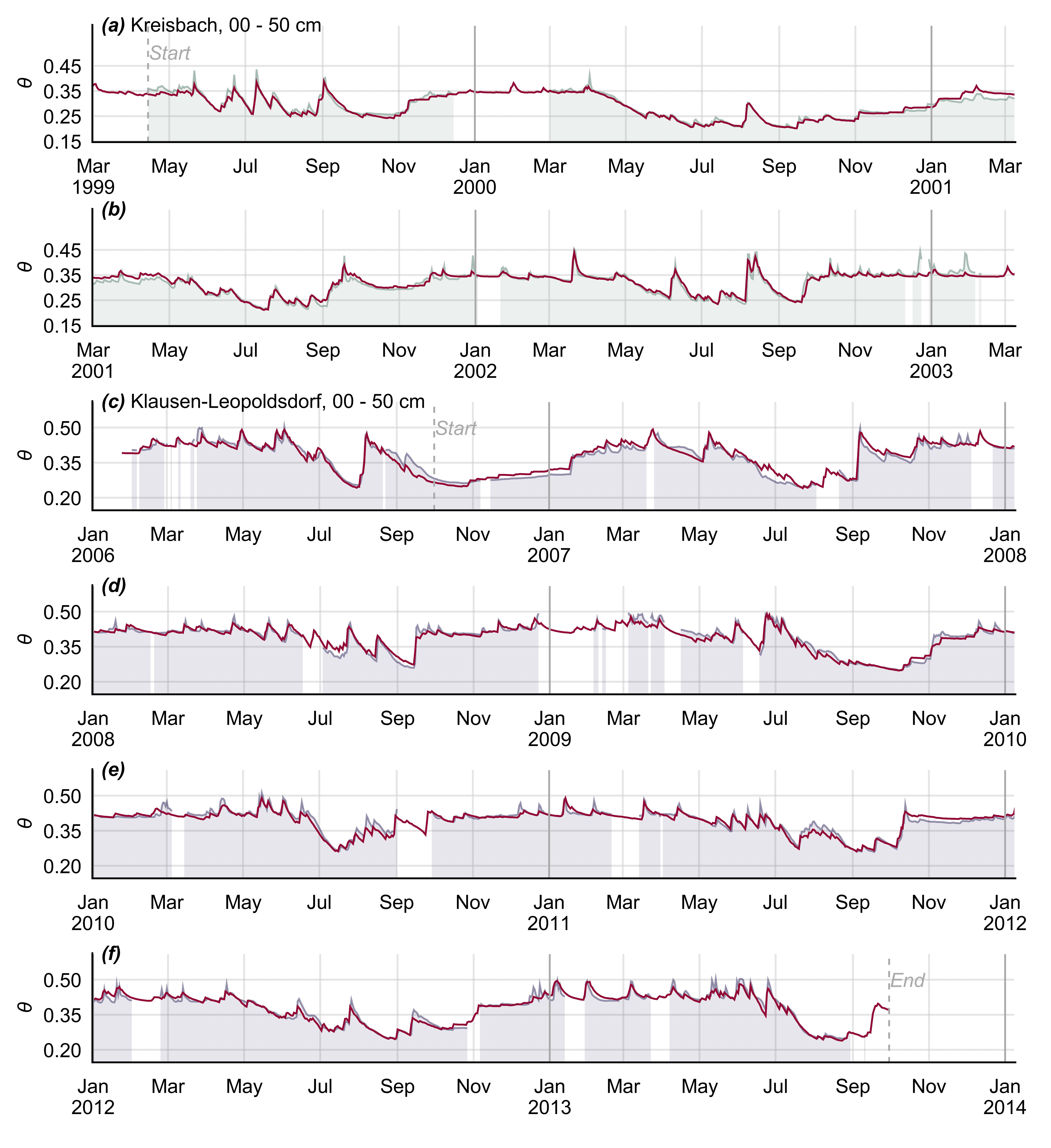
Fig. S6** Time series of observed (pale bands) and modeled (solid line) soil moisture. The calibration delivered an expedient estimate for 4 years of observed *θ* on the Kreisbach site (a, b). On the Klausen Leopoldsdorf site (c – f) the simulator was able to track *θ* over an almost seven-year period.

**
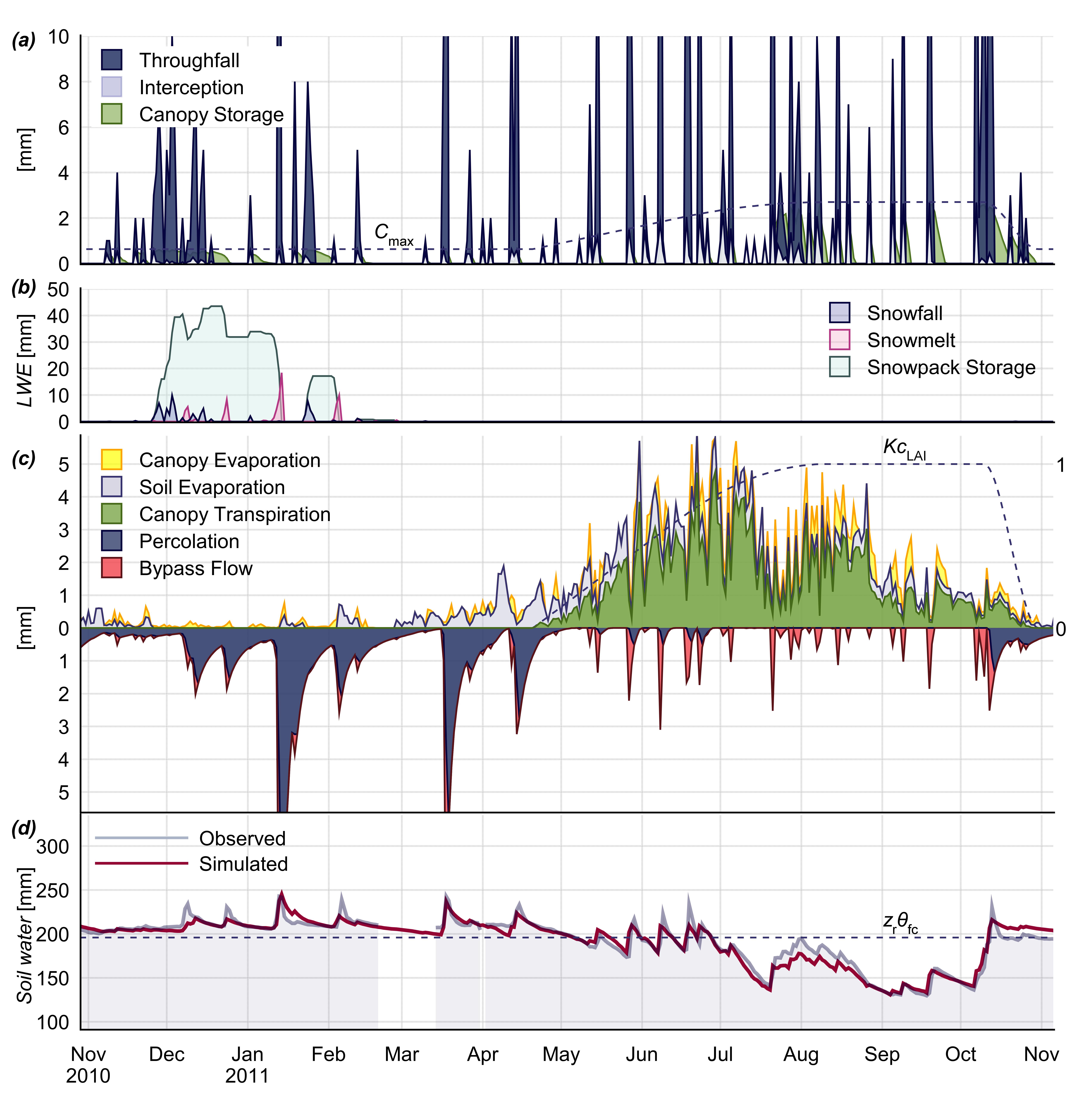
Fig. S7** Breakdown of daily modeled pools and fluxes for a one-year section of the Klausen-Leopoldsdorf plot. (a) Due to the nullity of the fog precipitation module on this site, the sum of throughfall and interception equals the observed precipitation. (c) Export fluxes: Gaseous and liquid exports are stated as positive and negative values, respectively. The high interception capacity of the canopy, combined with a high canopy specific crop coefficient (*Kc*_canopy_), sets canopy evaporation as the dominant pathway for gaseous losses. Compared to canopy evaporation and transpiration, the fraction soil evaporation is contributing to gaseous exports, is negligible. (d) Soil moisture, almost constantly above the field capacity (*θ*_fc_) during the dormant season, clearly states (c) percolation through the soil profile as a phenomenon of the cold part of the year.

**
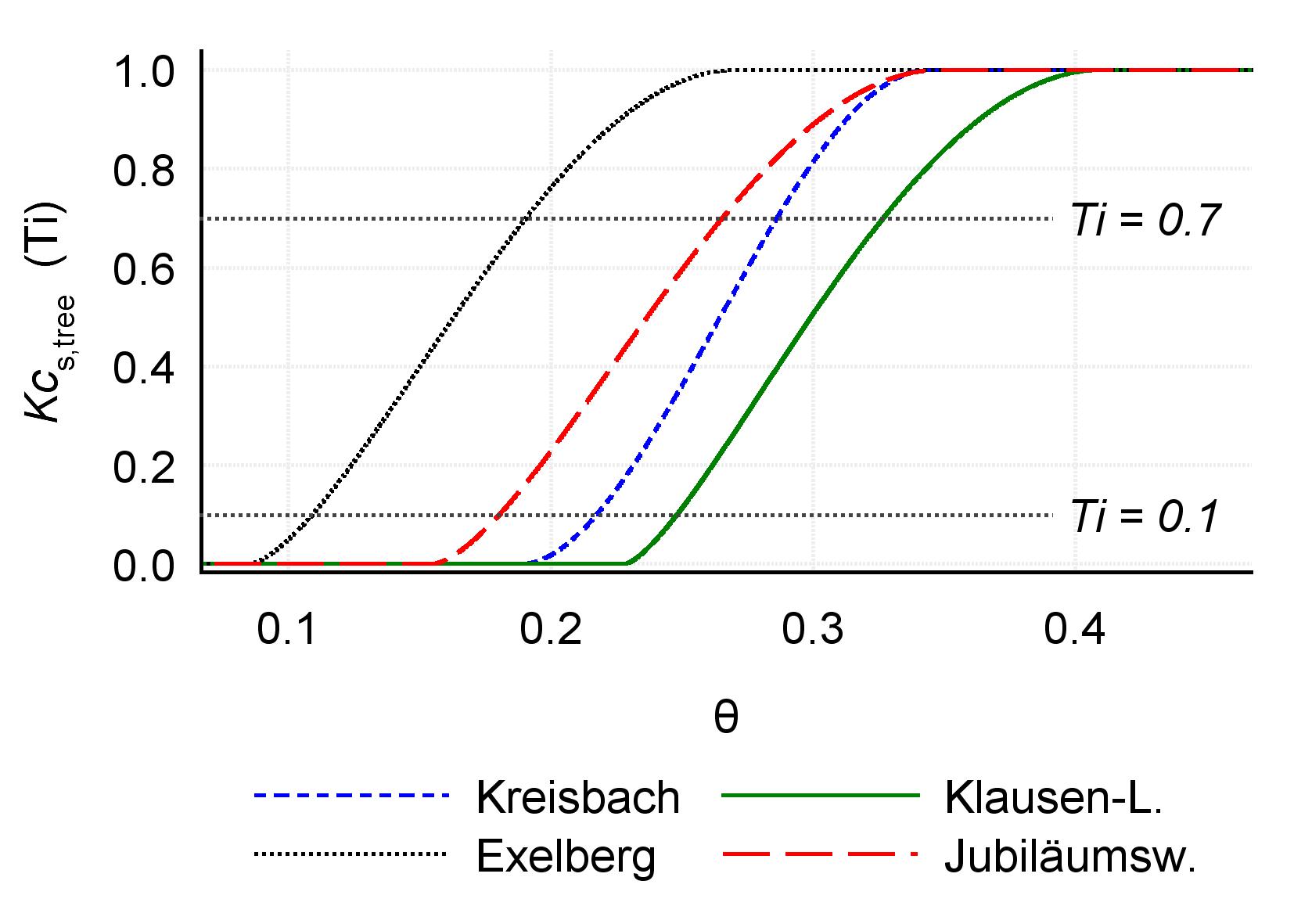
Fig. S8** The transpiration stress coefficient is dependent on soil moisture. The transition between *θ*_pwp_ and *θ^*^* is modeled utilizing the modified smoothstep function (Eq. A4). Severe water stress is assumed when Ti drops below 0.1. At the EX forest stand, this corresponds to relative soil moisture levels below ~11%.

**
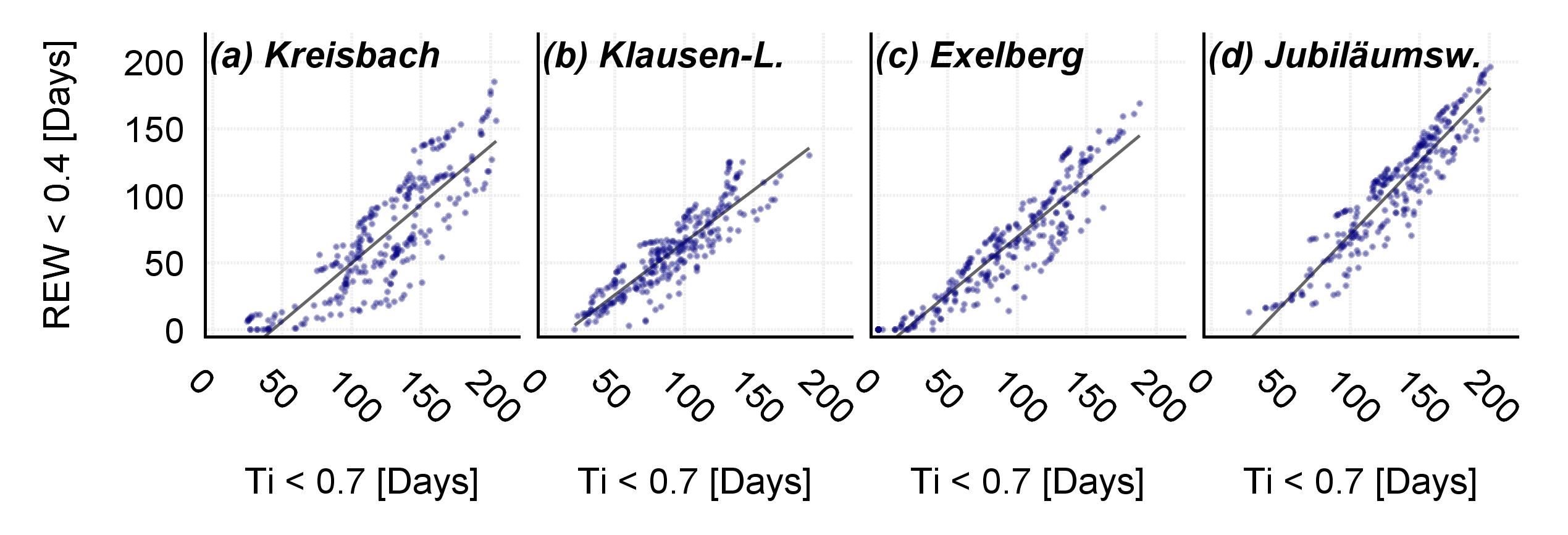
**

**Fig. S9** Site-wise comparison of water stress indices: Number of days per growing season, where actual transpiration falls below 70% of the potential evaporation vs. days per growing season, the relative extractable water soil water content (REW) falls below 0.4. The growing season is considered as period from the 25^th^ of March to the 11^th^ of November. Generally, both indices deliver similar results; the easterly sites (EX, JU) exhibit higher correlation.

**References**

Allen RG, Pereira LS, Raes D, Smith M (1998) Crop evapotranspiration-Guidelines for computing crop water requirements-FAO Irrigation and drainage paper 56 FAO, Rome 300:6541

Bastiaanssen WGM, Cheema MJM, Immerzeel WW, Miltenburg IJ, Pelgrum H (2012) Surface energy balance and actual evapotranspiration of the transboundary Indus Basin estimated from satellite measurements and the ETLook model Water Resour Res 48:W11512 doi:Doi 10.1029/2011wr010482

Baudena M, Bevilacqua I, Canone D, Ferraris S, Previati M, Provenzale A (2012) Soil water dynamics at a midlatitude test site: Field measurements and box modeling approaches J Hydrol 414:329-340 doi:DOI 10.1016/j.jhydrol.2011.11.009

Dolschak K, Gartner K, Berger TW (2015) A new approach to predict soil temperature under vegetated surfaces Modeling Earth Systems and Environment 1:1-14 doi:DOI 10.1007/s40808-015-0041-2

Federer C (1995) BROOK90: a simulation model for evaporation, soil water and streamflow, 3.23 edn. USDA Forest Service, Durham NH

Feiccabrino J, Lundberg A Precipitation phase discrimination in Sweden. In: Pomeroy J (ed) 65th Eastern Snow Conference, Fairlee (Lake Morey), Vermont, USA, 2008. pp 239-254

Hock R (2003) Temperature index melt modelling in mountain areas J Hydrol 282:104-115 doi:Doi 10.1016/S0022-1694(03)00257-9

Kumagai T, Yoshifuji N, Tanaka N, Suzuki M, Kume T (2009) Comparison of soil moisture dynamics between a tropical rain forest and a tropical seasonal forest in Southeast Asia: Impact of seasonal and year-to-year variations in rainfall Water Resour Res 45:W04413 doi:Doi 10.1029/2008wr007307

Laio F, Porporato A, Ridolfi L, Rodriguez-Iturbe I (2001) Plants in water-controlled ecosystems: active role in hydrologic processes and response to water stress - II. Probabilistic soil moisture dynamics Adv Water Resour 24:707-723 doi:Doi 10.1016/S0309-1708(01)00005-7

Rango A, Martinec J (1995) Revisiting the degree‐day method for snowmelt computations JAWRA Journal of the American Water Resources Association 31:657-669

Slinn WGN (1982) Predictions for Particle Deposition to Vegetative Canopies Atmos Environ 16:1785-1794 doi:Doi 10.1016/0004-6981(82)90271-2

van Dijk AIJM, Bruijnzeel LA (2001) Modelling rainfall interception by vegetation of variable density using an adapted analytical model. Part 1. Model description J Hydrol 247:230-238 doi:Doi 10.1016/S0022-1694(01)00392-4

Willen D, Shumway C, Reid J (1971) Simulation of daily snow water equivalent and melt Proceedings 1971 Western Snow Conference 39:1-8
