# Supplementary material for: The impact of rising temperatures on water balance and phenology of European beech (Fagus sylvatica L.) stands
Source: Model Earth Syst Environ. Author manuscript; Available in PMC 2019 Dec 1. (PMC6814441; doi:10.1007/s40808-019-00602-1)
Supplement: Appendix [file EMS84601-supplement-Appendix.pdf]

Appendix

To achieve sigmoid shape transitions of a variables (x) response (y) inside a window (x_0 to x_1), the 'smoothstep function' (compare Dolschak et al. 2015) was applied in several cases. y_0 and y_1 state left and right threshold responses, respectively. The variable x has to be normalized into an auxiliary variable (t) inside the interval 0–1:

$$t = \frac{x - x_0}{x_1 - x_0}. \tag{18}$$

The transition is described using a third order polynomial:

$$y = \begin{cases} y_0, & x \le x_0 \\ y_0 + (y_1 - y_0)(3t^2 - 2t^3), & x_0 < x < x_1 \\ y_1, & x \ge x_1 \end{cases}. \tag{19}$$

In the article the smoothstep function is stated as:

$$y = \text{smooth}(x, x_0, x_1, y_0, y_1). \tag{20}$$

To alter the shape of the transition, the latter function was modified, by introducing an exponent term:

$$y = \begin{cases} y_0, & x \le x_0 \\ y_0 + (y_1 - y_0)(3t^2 - 2t^3)^{\ln 0.5/\ln m}, & x_0 < x < x_1 \\ y_1, & x \ge x_1 \end{cases}. \tag{21}$$

The parameter m states the relative x position between x_0 and x_1 where the response (y) reaches 50% of the transition from y_0 to y_1. In the article the 'modified smoothstep function' is stated as:

$$y = \text{msmooth}(x, x_0, x_1, y_0, y_1, m). \tag{22}$$

The description of the air temperature dependent forcing of spring phenology uses a combination of a second and first order polynomial. The variable, which is being transformed, has to be normalized to the interval x_0 to x_1, using Eq. 18.

$$y = y_0 + (y_1 - y_0)\begin{cases} 0, & x \le x_0 \\ t,^2 & x_0 < x < x_1 \\ 2t - 1, & x \ge x_1 \end{cases} \tag{23}$$

In the paper, the function, which is optically resembling the shape of a hockey stick, is stated as:

$$y = \text{hockey}(x, x_0, x_1, y_0, y_1). \tag{24}$$

We calculate precipitation interception using a function, based on the Langmuir sorption isotherm. I states interception, C_{max} the maximum charge of the canopy, K_{i} the shape parameter and T throughfall:

$$I = \frac{K_{\text{i}} C_{\text{max}} T}{1 + K_{\text{i}} T}. \tag{25}$$

Substituting Interception with the difference of precipitation (R) and throughfall yields:

$$R - T = \frac{K_{\text{i}} C_{\text{max}} T}{1 + K_{\text{i}} T}. \tag{26}$$

Avoiding the intricacies of an implicit formulation, we solve the equation for throughfall:

$$T = \frac{\sqrt{K_{\text{i}}^2 (C_{\text{max}} - R)^2 + 2K_{\text{i}}(C_{\text{max}} + R) + 1} + K_{\text{i}}(R - C_{\text{max}}) - 1}{2K_{\text{i}}}. \tag{27}$$

In the article this 3-argument function is stated as:

$$T = \text{tru}(R, C_{\text{max}}, K_{\text{i}}). \tag{28}$$
